# ER-associated RNA silencing promotes ER quality control

**DOI:** 10.1038/s41556-022-01025-4

**Published:** 2022-12-05

**Authors:** Sotirios Efstathiou, Franziska Ottens, Lena-Sophie Schütter, Sonia Ravanelli, Nikolaos Charmpilas, Aljona Gutschmidt, Jérémie Le Pen, Niels H. Gehring, Eric A. Miska, Jorge Bouças, Thorsten Hoppe

**Affiliations:** 1 Institute for Genetics, University of Cologne, Cologne, Germany; 2 Cologne Excellence Cluster on Cellular Stress Responses in Aging-Associated Diseases (CECAD), University of Cologne, Cologne, Germany; 3 Laboratory of Virology and Infectious Disease, The Rockefeller University, New York, NY USA; 4 Wellcome Trust/Cancer Research UK Gurdon Institute, University of Cambridge, Cambridge, UK; 5 Department of Genetics, University of Cambridge, Cambridge, UK; 6 Center for Molecular Medicine Cologne (CMMC), Faculty of Medicine and University Hospital of Cologne, Cologne, Germany; 7 Wellcome Sanger Institute, Wellcome Trust Genome Campus, Cambridge, UK; 8 Bioinformatics Core Facility, Max Planck Institute for Biology of Aging, Cologne, Germany; 9 Present Address: BioNTech SE, Mainz, Germany

**Keywords:** ER-associated degradation, RNAi, Endoplasmic reticulum

## Abstract

The endoplasmic reticulum (ER) coordinates mRNA translation and processing of secreted and endomembrane proteins. ER-associated degradation (ERAD) prevents the accumulation of misfolded proteins in the ER, but the physiological regulation of this process remains poorly characterized. Here, in a genetic screen using an ERAD model substrate in *Caenorhabditis elegans*, we identified an anti-viral RNA interference pathway, referred to as ER-associated RNA silencing (ERAS), which acts together with ERAD to preserve ER homeostasis and function. Induced by ER stress, ERAS is mediated by the Argonaute protein RDE-1/AGO2, is conserved in mammals and promotes ER-associated RNA turnover. ERAS and ERAD are complementary, as simultaneous inactivation of both quality-control pathways leads to increased ER stress, reduced protein quality control and impaired intestinal integrity. Collectively, our findings indicate that ER homeostasis and organismal health are protected by synergistic functions of ERAS and ERAD.

## Abstract (web summary)

Efstathiou et al. describe an Argonaute-dependent endoplasmic reticulum (ER)-associated RNA silencing pathway that acts together with ER-associated protein degradation to preserve ER homeostasis and function.

## Main

The endoplasmic reticulum (ER) coordinates translation, folding and maturation of luminal, secreted and transmembrane proteins within the endomembrane system. Challenging the protein folding capacity of the ER by increased protein import or drug-induced dysfunction results in ER stress, which activates a series of conserved protein quality-control pathways that together constitute the unfolded protein response of the ER (UPR^ER^)^1,2^. ER stress signalling is essential for organismal development and has been associated with many pathological states, including metabolic, neurologic and inflammatory diseases^3,4^.

Upon ER stress, the inositol-requiring enzyme (IRE1), a transmembrane protein kinase and endoribonuclease, promotes the unconventional splicing of the XBP1-encoding messenger RNA precursor, leading to the translation of the transcription factor XBP1s (refs. ^5–7^). One of the molecular mechanisms controlled by XBP1s is the ER-associated protein degradation (ERAD) pathway. ERAD targets ER-resident proteins for selective degradation by the ubiquitin/proteasome system^8^. Under stress conditions, ERAD prevents the accumulation of misfolded proteins through the luminal ER chaperone BiP and glycoprotein-binding lectins, which cooperate with the Sel1L–Hrd1 protein complex. Sel1L is an obligatory co-factor for the ER membrane spanning E3 ligase Hrd1, which ubiquitylates retro-translocated proteins for proteasomal turnover in the cytosol. In mammalian cells, IRE1 overexpression initiates the cleavage of particular mRNAs in a process termed regulated IRE1-dependent mRNA decay^9,10^. However, the functional relevance of this mechanism remains largely unclear, since mRNA levels of most regulated IRE1-dependent mRNA decay targets do not notably change even under ER stress conditions^11^.

In this Article, we investigated the physiological regulation of ERAD and discovered an mRNA silencing mechanism that ensures ER homeostasis and function. In response to ER stress, the conserved Argonaute protein RDE-1/AGO2 mediates the bulk degradation of ER-associated mRNAs to ameliorate ER overload. This protective mechanism that we named ER-associated RNA silencing (ERAS) acts together with ERAD to maintain ER homeostasis and tissue integrity. Our work shows that ER-targeted mRNAs and proteins need to be actively degraded to allow the ER to cope with stress and maintain the essential function of this organelle as the cellular sorting centre.

## Results

### The exo-RNAi pathway is linked to ERQC

**Fig. 1:**
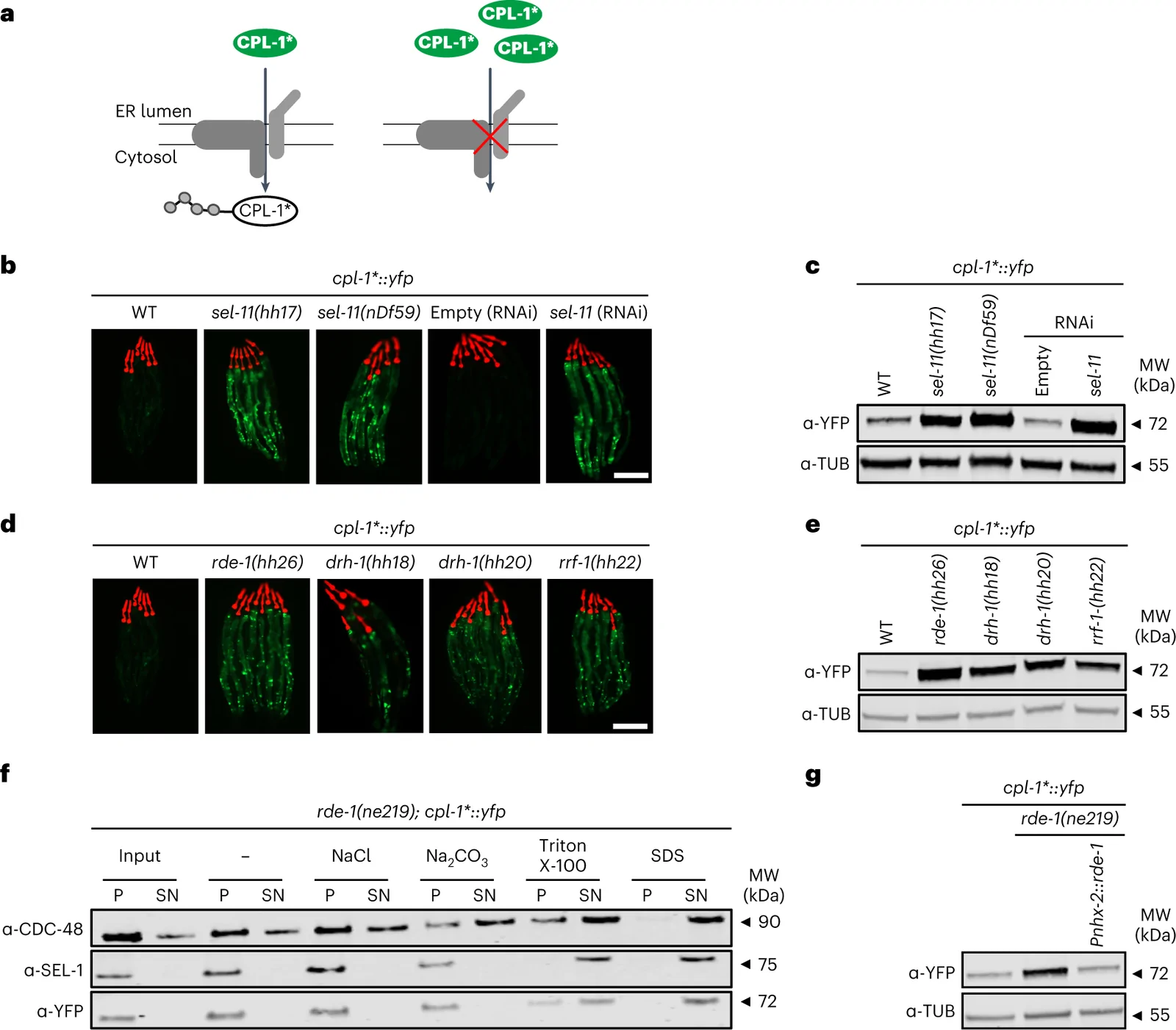
A role for exo-RNAi in ER homeostasis. **a**, Turnover of the model substrate CPL‑1* via ERAD. **b**, Fluorescence images of worms expressing *cpl*‑1* with the indicated mutations and RNAi (empty control (Empty)) for the ERAD factor *sel-11*. **c**, Western blot of worm lysates corresponding to images shown in **b**, detecting CPL-1* (YFP) and tubulin (TUB). **d**, Fluorescence images of worms expressing cpl-1* with mutations in the exo-RNAi pathway. **e**, Western blot of worm lysates corresponding to the samples in **d**, detecting CPL-1* (YFP) and tubulin (TUB). **f**, Cellular fractionation of microsomes. Western blot detecting CPL-1* (YFP), CDC-48 (CDC-48) and SEL-1 (SEL-1). Pellet (P) and supernatant (SN) fractions of rde-1(ne219); cpl-1* worm lysates treated with the indicated chemicals after subcellular fractionation. **g**, Western blot of worm lysates detecting CPL-1* (YFP) and tubulin (TUB) in *rde-1(ne219)* worms and animals with intestinal *Pnhx-2*::*rde-1* rescue. In **b** and **d**, pharyngeal expression of *Pmyo-2::mCherry* serves as transgenic marker. Scale bar, 200 µm. MW, molecular weight. Unprocessed blots are available in the source data. Source data

**Extended Data Fig. 1:**
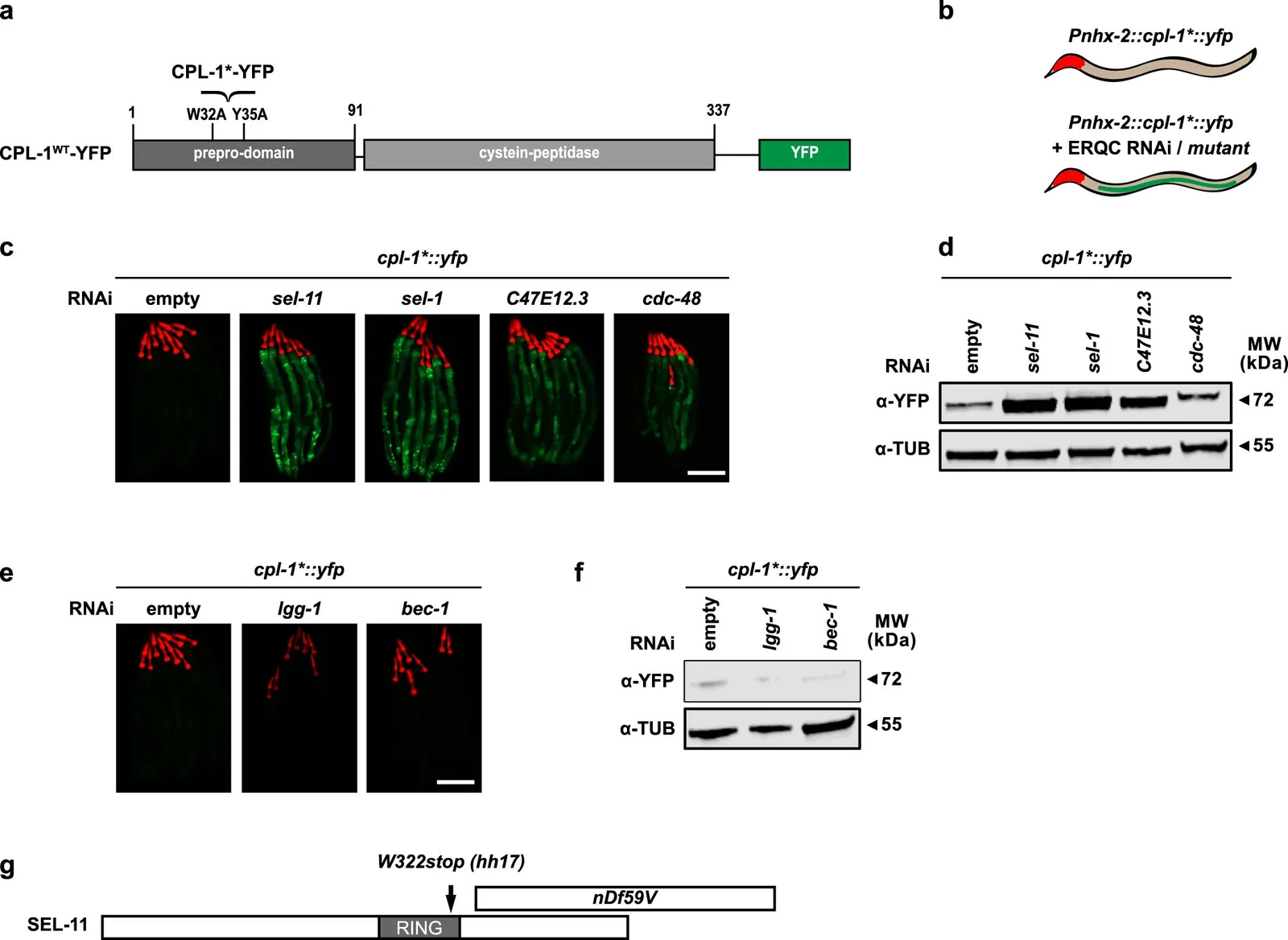
CPL-1* model substrate to monitor ER quality control in *C. elegans*. (**a**) Schematic overview of mutations inserted into the CPL-1^WT^ encoding transgene for expression of the CPL-1* ERAD substrate. (**b**) Schematic overview of the CPL-1* model substrate for studying ERQC. (**c**) Fluorescence images of worms with mutations in ERAD components expressing cpl-1*; C47E12.3 (EDEM-1 homologue), empty vector (empty). (**d**) Western blot of worm lysates corresponding to samples shown in (c), detecting CPL-1* (YFP) and tubulin (TUB). (**e**) Fluorescence images of worms expressing cpl-1* with defects in autophagy. (**f**) Western blot of worm lysates corresponding to the samples in (e), detecting CPL-1* (YFP) and tubulin (TUB). (**g**) Schematic overview of different *sel-11* alleles used in this study; *nDf59*: deletion allele, *hh17*: Introduction of a stop codon at amino acid position 322 of the SEL-11 protein. [(c), (e)] Pharyngeal expression of *Pmyo-2::mCherry* serves as transgenic marker. Scale bar: 200 µm. WT: wild-type. Unprocessed blots are available in the source data. Source data

To investigate physiological ER quality control (ERQC) mechanisms, we examined the ERAD substrate protein CPL-1*::YFP (CPL-1*) in *Caenorhabditis elegans*. CPL-1*, a misfolding mutant form of the lysosomal cathepsin L-like peptidase, accumulates in the ER lumen when ERAD is impaired (Fig. 1a and Extended Data Fig. 1a)^12,13^. We stably expressed CPL-1* in intestinal cells, which are enriched in rough ER and secretory vesicles (Extended Data Fig. 1b)^13^. Knockdown of the E3 ligase SEL-11 (Hrd1 orthologue), its adaptor protein SEL-1 (Sel1L orthologue), the lectin C47E12.3 (EDEM1 orthologue) or the chaperone CDC-48 resulted in increased YFP levels, validating CPL-1* as a bona fide ERAD substrate (Extended Data Fig. 1c,d). In contrast, depletion of the autophagy regulators LGG-1 (LC3 orthologue) or BEC-1 (Beclin-1 orthologue) did not change CPL-1* protein levels (Extended Data Fig. 1e,f).

**Extended Data Fig. 2:**
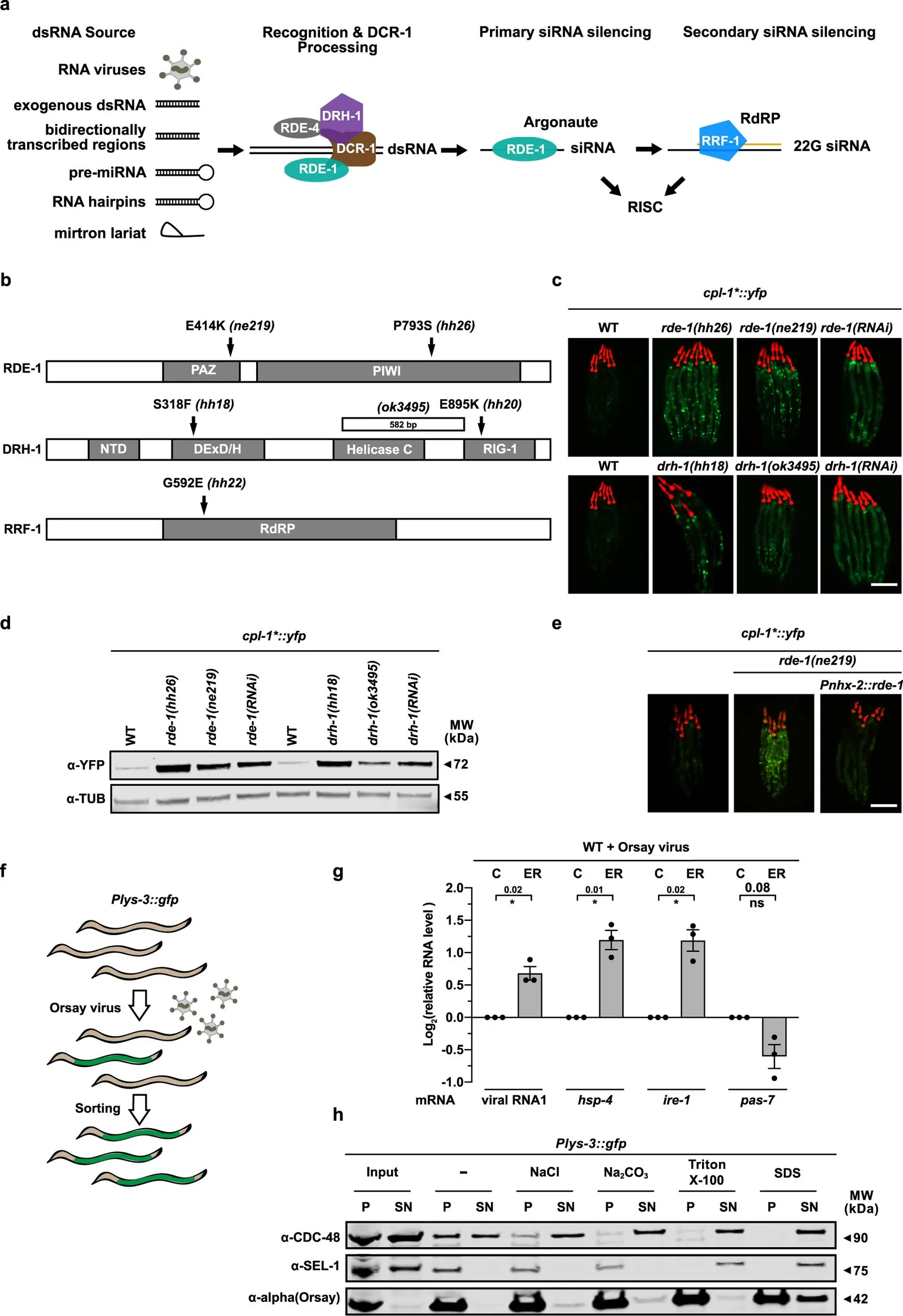
Exogenous RNAi pathway mutants accumulate the CPL-1* protein. (**a**) Scheme of the exogenous RNAi pathway, detecting double stranded RNA (dsRNAs) species from various sources. RDE-4 and DRH-1 are involved in dsRNA recognition, whereas DCR-1 cleaves these RNAs into primary small interfering or micro RNAs (siRNAs/miRNA). These small RNA species are either used for primary siRNA silencing by RDE-1 or engage RDE-1-dependently in amplification by the RNA-dependent RNA polymerase (RdRP) RRF-1. RRF-1 generates secondary siRNAs for potent secondary siRNA silencing. Eventually, siRNAs and their complementary target mRNAs are loaded into the RNA-induced silencing complex (RISC) mediating target mRNA silencing. (**b**) Schematic overview of loss-of-function alleles of *rde-1, drh-1, and rrf-1*. *ne219, hh26, hh18, hh20, hh22*: amino acid substitutions; *ok3495*: deletion allele. NTD: N-terminal domain, RdRP: RNA-dependent RNA polymerase. (**c**) Fluorescence images of worms defective in the exogenous RNAi pathway expressing cpl-1*. (**d**) Western blot of worm lysates corresponding to samples shown in (c), detecting CPL-1* (YFP) and tubulin (TUB). (**e**) Fluorescence images of *rde-1(ne219)* worms and animals with intestinal *Pnhx-2*::*rde-1* rescue expressing cpl-1*. (**f**) Schematic overview of Orsay virus infection in worms expressing the viral infection reporter *Plys-3::gfp*. Infected worms can be detected and sorted by the green fluorescent signal. (**g**) Cellular fractionation of cytosolic (C) and ER fraction (ER) with subsequent RNA purification. Relative mRNA level measured by qRT-PCR. (**h**) Cellular fractionation of microsomes. Western blot detecting SEL-1 (SEL-1), CDC-48 (CDC-48), and Orsay virus alpha capsid protein (alpha (Orsay)). Pellet (P) and supernatant (SN) fractions of *Plys-3::gfp* worm lysates treated with the indicated chemicals after subcellular fractionation. [(c), (e)] Pharyngeal expression of *Pmyo-2::mCherry* serves as transgenic marker. Scale bar: 200 µm. (g) was analyzed by unpaired two-tailed t-tests. Values are depicted as mean ± SEM generated from n = 3 independent experiments, *p < 0.05, ns p > 0.05. WT: wild-type. Source numerical data and unprocessed blots are available in source data. Source data

We performed a forward genetic screen using ethyl methane sulfonate (EMS)-induced mutagenesis and isolated YFP-positive mutants with increased CPL-1* levels (Fig. 1a and Extended Data Fig. 1a,b). Whole-genome sequencing and genetic mapping of selected mutants identified known components of ERAD, including *sel-11* (Fig. 1b,c and Extended Data Fig. 1g)^14^. The screen also revealed that *rde-1(hh26)*, *rde-1(ne219)*, *drh-1(hh18)*, *drh-1(hh20)*, *drh-1(ok3495)* and *rrf-1(hh22)* loss-of-function (LOF) mutants (Extended Data Fig. 2b), or their RNA interference (RNAi) depletion, robustly increased CPL-1* protein levels, as seen in *sel-11(hh17)* (Fig. 1d,e and Extended Data Fig. 2c,d). CPL-1* co-fractionated with SEL-1 in ER-derived microsomes of *rde-1(ne219)* worms (Fig. 1f), indicating that the CPL-1* protein accumulated in the ER fraction. Intestinal expression of *rde-1* rescued the accumulation of CPL-1* in the *rde-1(ne219)* mutant (Fig. 1g and Extended Data Fig. 2e). Thus, our identification of *rde-1, drh-1* and *rrf-1* as factors in CPL-1* accumulation suggested an involvement in ERQC.

RNAi defective (RDE-1), dicer-related helicase (DRH-1) and RNA-dependent RNA polymerase (RRF-1) are all components of the exogenous RNAi (exo-RNAi) pathway that mediates the degradation of viral RNAs (Extended Data Fig. 2a)^15^. Many viruses use the ER for their replication and also manipulate factors localized in the ER, such as ERAD, to their advantage^16^. We wanted to know whether Orsay virus, which infects *C. elegans* and is targeted by exo-RNAi^17,18^, also has ER association. To this end, we used a viral infection transcriptional reporter^19^ that allowed us to enrich worms infected with Orsay virus (Extended Data Fig. 2f). Like the CPL-1* protein, cellular fractionation assays showed that Orsay virus RNA1 and its capsid protein α are enriched in the ER fraction, suggesting that virus replication and exo-RNAi function at the ER membrane (Extended Data Fig. 2g,h).

### Exo-RNAi degrades CPL-1* substrate-encoding mRNA

**Fig. 2:**
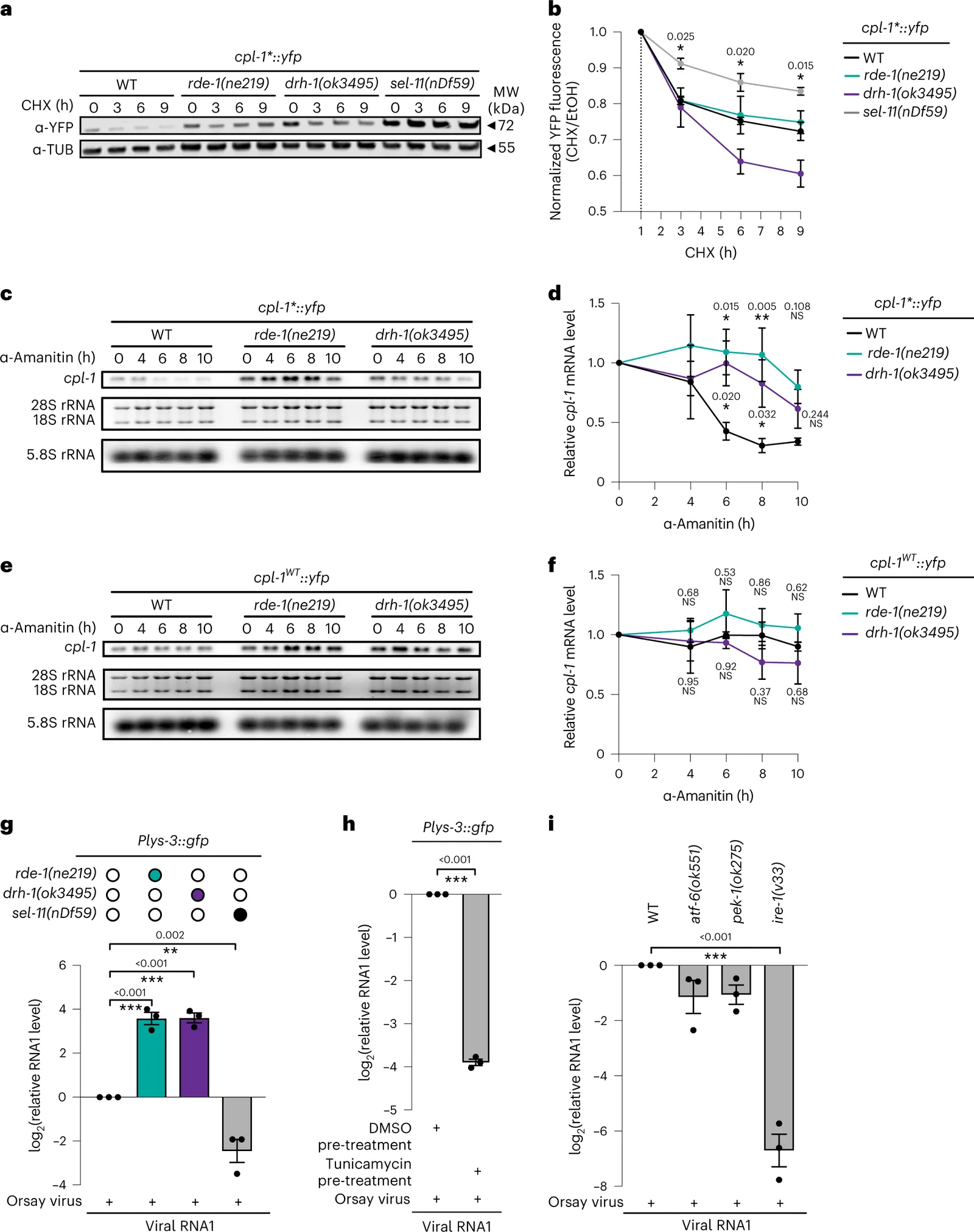
Post-transcriptional regulation of ERAD substrate and viral RNA level. **a**,**b**, CHX chase assay to assess CPL-1* protein stability: western blot of lysates from cpl-1* expressing worms with indicated mutations detecting CPL-1* (YFP) and tubulin (TUB) over 9 h of CHX treatment (**a**); BioSorter quantification of YFP fluorescence in vehicle (EtOH)- and CHX-treated cpl-1*-expressing worms (**b**). CHX/EtOH ratios were normalized to the 1 h timepoint of the respective genotype. **c**, α-Amanitin chase assay to monitor *cpl-1* mRNA level in *rde‑1(ne219)* and *drh‑1(ok3495)* mutant worms expressing cpl-1*. Northern blot of purified RNA from animals expressing cpl-1*, detecting *cpl-1* mRNA and 5.8S rRNA. 28S and 18S rRNA served as agarose gel loading control. **d**, Quantification of *cpl-1* mRNA level corresponding to the data shown in **c**, relative to 5.8 S rRNA. **e**, α-Amanitin chase assay to monitor *cpl-1* mRNA level in *rde‑1(ne219)* and *drh‑1(ok3495)* mutant worms expressing *cpl-1*^*WT*^. Northern blot of purified RNA from animals expressing *cpl-1*^*WT*^, detecting *cpl-1* mRNA and 5.8 S rRNA. 28S and 18S rRNA served as agarose gel loading control. **f**, Quantification of *cpl-1* mRNA level corresponding to the data shown in **e**, relative to 5.8S rRNA. **g**, Viral RNA1 level measured by qRT–PCR in worms infected with the Orsay virus. **h**, Viral RNA1 level measured by qRT–PCR in worms pre-treated with vehicle (DMSO) or tunicamycin before viral infection. Data are relative to 18S rRNA. **i**, Viral RNA1 level measured by qRT–PCR in worms, defective for the UPR^ER^ pathway. In **b**, **d** and **f**–**i**, Values are depicted as mean ± standard error of the mean (s.e.m.) generated from *n* = 3 independent experiments, *P < 0.05; **P < 0.01; ***P < 0.001; NS, *P* > 0.05. Data were analysed by two-way ANOVA with Sidak’s multiple comparison test. Source numerical data and unprocessed blots are available in source data. Source data

**Extended Data Fig. 3:**
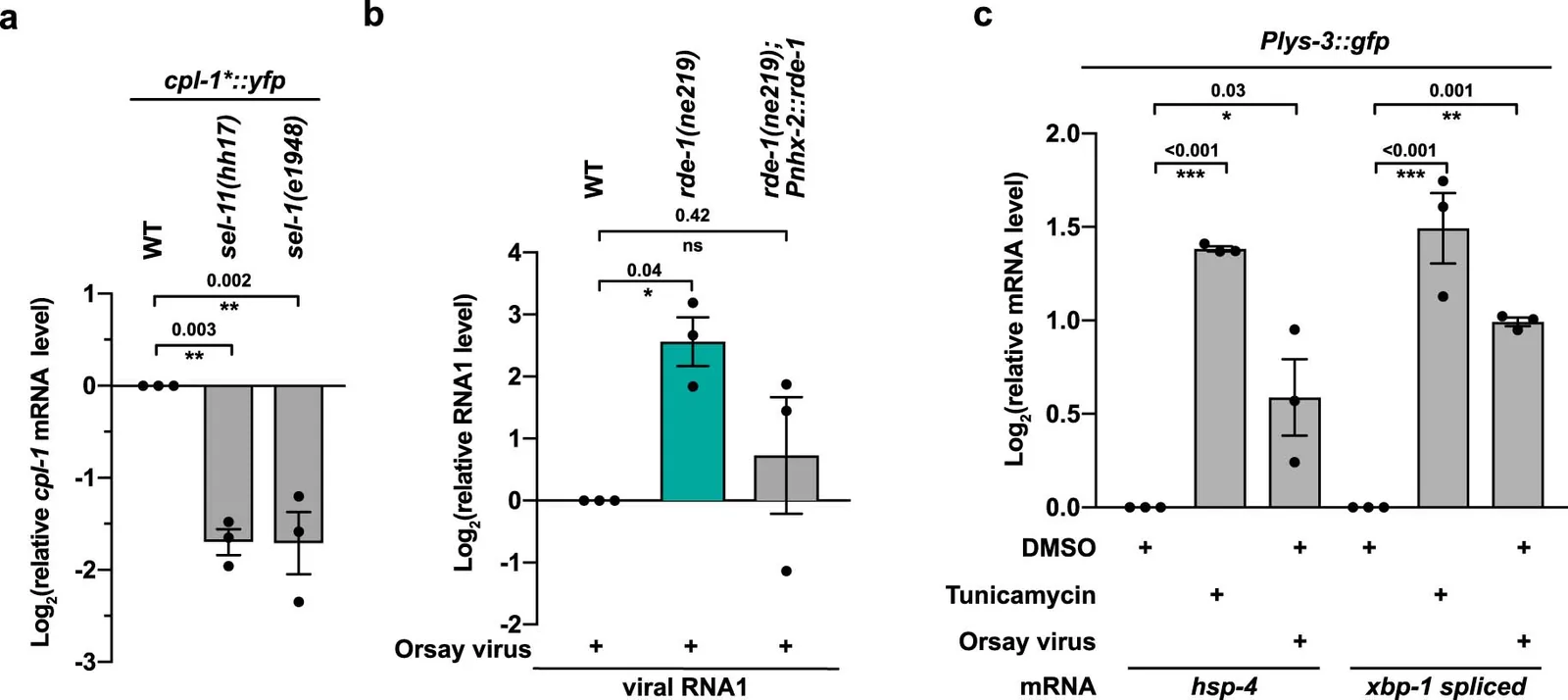
The exogenous RNAi pathway degrades viral RNA. (**a**) *cpl-1* mRNA level measured by qRT-PCR in worms depleted of the ERAD components *sel-1* or *sel-11*. (**b**) Viral RNA1 level measured by qRT-PCR in *rde-1(ne219)* worms and animals with intestinal *Pnhx-2*::*rde-1* expression. (**c**) *hsp-4* and spliced *xbp-1* mRNA level measured by qRT-PCR in worms infected with the Orsay virus or treated with tunicamycin. [(a)-(c)] were analyzed by one-way ANOVA with Sidak’s multiple comparison test. Data are relative to 18 S rRNA. Values are depicted as mean ± SEM generated from n = 3 independent experiments, *p < 0.05, **p < 0.01, ***p < 0.001, ns p > 0.05. WT: wild-type. Source numerical data are available in source data. Source data

To examine the role of the exo-RNAi machinery in ERQC we monitored CPL-1* protein turnover over a period of 9 h after blocking translation with cycloheximide (CHX). While CPL-1* degradation was significantly impaired in the ERAD-defective mutant *sel-11(nDf59)*, the *rde-1(ne219)* and *drh-1(ok3495)* mutants were not impaired in substrate protein degradation (Fig. 2a,b), indicating that its accumulation in *rde-1* and *drh-1* (Fig. 1d,e) was not linked to ERAD-mediated post-translational control. RDE-1 belongs to the Argonaute protein family^20^ and contains a PIWI/Argonaute/Zwille (PAZ) domain and a PIWI domain with endonucleolytic activity^21^, which is required for RNAi^22^. Given that RDE-1 and the DRH-1 RNA helicase form part of the RNA-induced silencing complex, which participates in target RNA degradation (Extended Data Fig. 2a), we wondered if they directly targeted the transgenic *cpl-1* mRNA. The *rde-1(ne219)* LOF lesion converts a conserved glutamate to lysine within the PAZ domain, which binds the 3′ end of small interfering RNAs (siRNAs)^22^, abrogating its RNA silencing function (Extended Data Fig. 2b). Thus, we reasoned that the exo-RNAi pathway might affect CPL-1* protein levels via post-transcriptional regulation of transgenic cpl-1* mRNA turnover. To test this idea, we blocked transcription using α-amanitin and measured cpl-1* mRNA levels over 10 h. We observed rapid mRNA turnover in wild-type (WT) animals, but increased stability in *rde-1(ne219)* and *drh-1(ok3495)* mutant worms (Fig. 2c,d). Intriguingly, we found decreased cpl-1* mRNA baseline levels in the ERAD-defective *sel-11* and *sel-1* mutants (Extended Data Fig. 3a), suggesting a link between ERQC and mRNA turnover. To further investigate the interplay between ERAD and post-transcriptional regulation of mRNA turnover by exo-RNAi, we analysed *cpl-1* mRNA levels in worms expressing the natively folded wild-type CPL-1 (CPL-1^WT^) protein. In contrast to the rapid degradation of the cpl-1* transcript, *cpl-1*^*WT*^ mRNA remained unchanged in the absence of functional RDE-1 or DRH-1 (Fig. 2e,f), suggesting that the exo-RNAi pathway predominantly targets the cpl-1* mRNA.

As both CPL-1 and viral components are localized at the ER (Fig. 1f and Extended Data Fig. 2f–h), we examined the effects of ER homeostasis on Orsay virus infection in *C*. *elegans*. Consistent with the known anti-viral function of exo-RNAi^17,18^, *rde-1* and *drh-1* mutants showed increased levels of viral RNA1 upon infection with Orsay virus (Fig. 2g), which was rescued by intestinal *rde-1* expression (Extended Data Fig. 3b)^18,23–26^. Viral infection induced expression of the ER-resident chaperone *hsp-4* (BiP orthologue) and triggered splicing of *xbp-1* (Extended Data Fig. 3c). In addition, we found that viral RNA1 levels decreased sharply in worms pre-treated with the ER stressor tunicamycin before virus infection, indicating increased viral RNA turnover upon ER stress (Fig. 2h). The ERAD *sel-11* mutant also exhibited decreased RNA1 levels (Fig. 2g), as did the *atf-6*, *pek-1* and *ire-1* mutants, which have defects in UPR^ER^ regulation (Fig. 2i), suggesting a functional link between ER fidelity and viral RNA degradation. Our results suggest that ER stress triggers the degradation of ER-associated mRNAs and viral RNAs via the exo-RNAi machinery.

### mRNA turnover is linked to ER stress

**Fig. 3:**
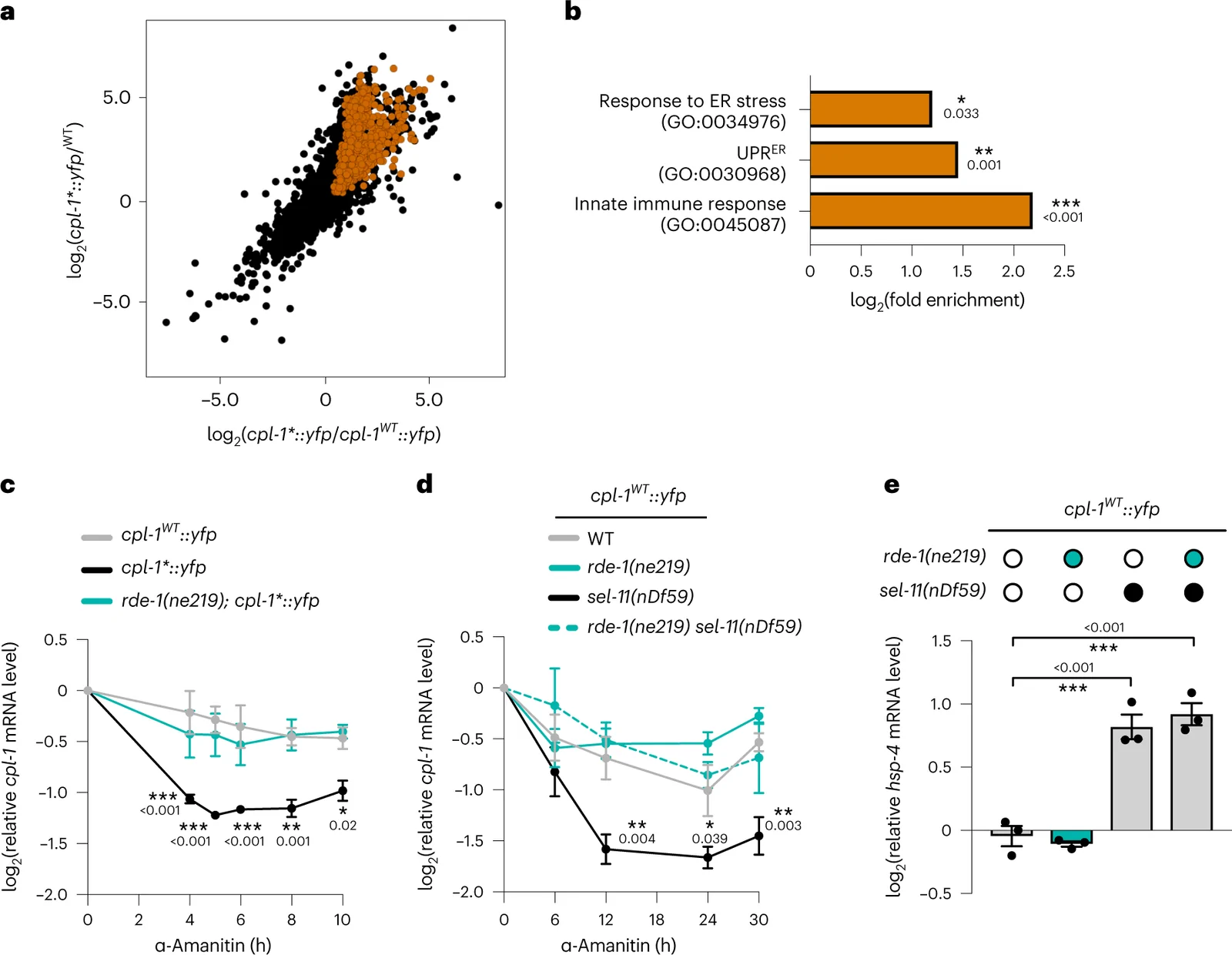
RDE-1-dependent RNA turnover is affected by ER stress. **a**, RNA sequencing results of cpl-1* expressing worms compared with WT and *cpl-1*^*WT*^ expressing worms, respectively. The cut-off for filtering of upregulated constructs was performed with *P* ≤ 0.05 (brown spheres). **b**, GO term enrichment for biological processes of the transcripts identified in **a** (brown). Only transcripts that were upregulated in both datasets shown in **a** were selected for analysis, resulting in 996 upregulated transcripts. **c**,**d**, α-Amanitin chase assays for *cpl-1*^*WT*^ and cpl-1* mRNA from mutant animals for ERAD and/or ERAS expressing *cpl-1*^*WT*^ or cpl-1* mRNA measured by qRT–PCR. Data relative to 18S rRNA, *cdc-42* and *pmp-3* mRNA. **e**, *hsp-4* mRNA level measured by qRT-PCR at the 0 h timepoint corresponding to the samples shown in **d**. Data relative to *cdc-42* and *pmp-3* mRNA. In **c** and **d**, data were analysed by two-way ANOVA with Dunnett’s multiple comparison test. In **e**, data were analysed by one-way ANOVA with Sidak’s multiple comparison test. In **c**–**e**, values are depicted as mean ± s.e.m. generated from *n* = 3 independent experiments, *P < 0.05; **P < 0.01; ***P < 0.001; NS, *P* > 0.05. In **b**, raw *P* values were corrected for FDR. *FDR <0.05; **FDR <0.01; ***FDR <0.001; NS, FDR >0.05. Source numerical data are available in source data. Source data

To investigate the signalling mechanism that activates exo-RNAi, we analysed the transcriptional landscape of worms expressing cpl-1* and compared them with *cpl-1*^*WT*^ and wild-type worms by RNA sequencing. Gene Ontology (GO) term enrichment analysis of the significantly upregulated transcripts identified genes involved in UPR^ER^, response to ER stress, and innate immunity (Fig. 3a,b), suggesting that overexpression and misfolding of CPL-1* increased protein folding load and ERQC requirements of the ER and induced ER stress^13,27,28^.

We hypothesized that increased ER stress caused by cpl-1* expression or *sel-11* deficiency determines substrate mRNA stability (Fig. 3a,b and Extended Data Fig. 3a), which could also explain the previously observed degradation of cpl-1* but not *cpl-1*^*WT*^ mRNA (Fig. 2c–f). To understand the relationship between ER stress and post-transcriptional regulation of mRNA turnover by exo-RNAi, we directly compared *cpl-1* mRNA levels in worms expressing either wild-type CPL-1 (CPL-1^WT^) or the misfolding CPL-1* substrate protein. Quantitative real-time polymerase chain reaction qRT–PCR showed that *cpl-1*^*WT*^ mRNA levels were stable for 10 h, whereas cpl-1* mRNA decreased rapidly (Fig. 3c). In *rde-1* mutant worms, the rapid decay of cpl-1* mRNA was suppressed (Fig. 3c), whereas the stability of *cpl-1*^*WT*^ mRNA was unchanged in the absence of functional RDE-1 (Fig. 3d). Strikingly, RDE-1-mediated degradation of *cpl-1*^*WT*^ transcripts can be triggered in the ERAD-defective *sel-11* mutant which shows increased ER stress, as observed by *hsp-4* expression levels (Fig. 3d,e). These data suggest that induction of ER stress makes *cpl-1*^*WT*^ mRNA an optional target of the exo-RNAi pathway. Thus, RDE-1-dependent mRNA turnover is coupled to ER homeostasis.

### RDE-1 regulates the turnover of ER-associated mRNAs

**Fig. 4:**
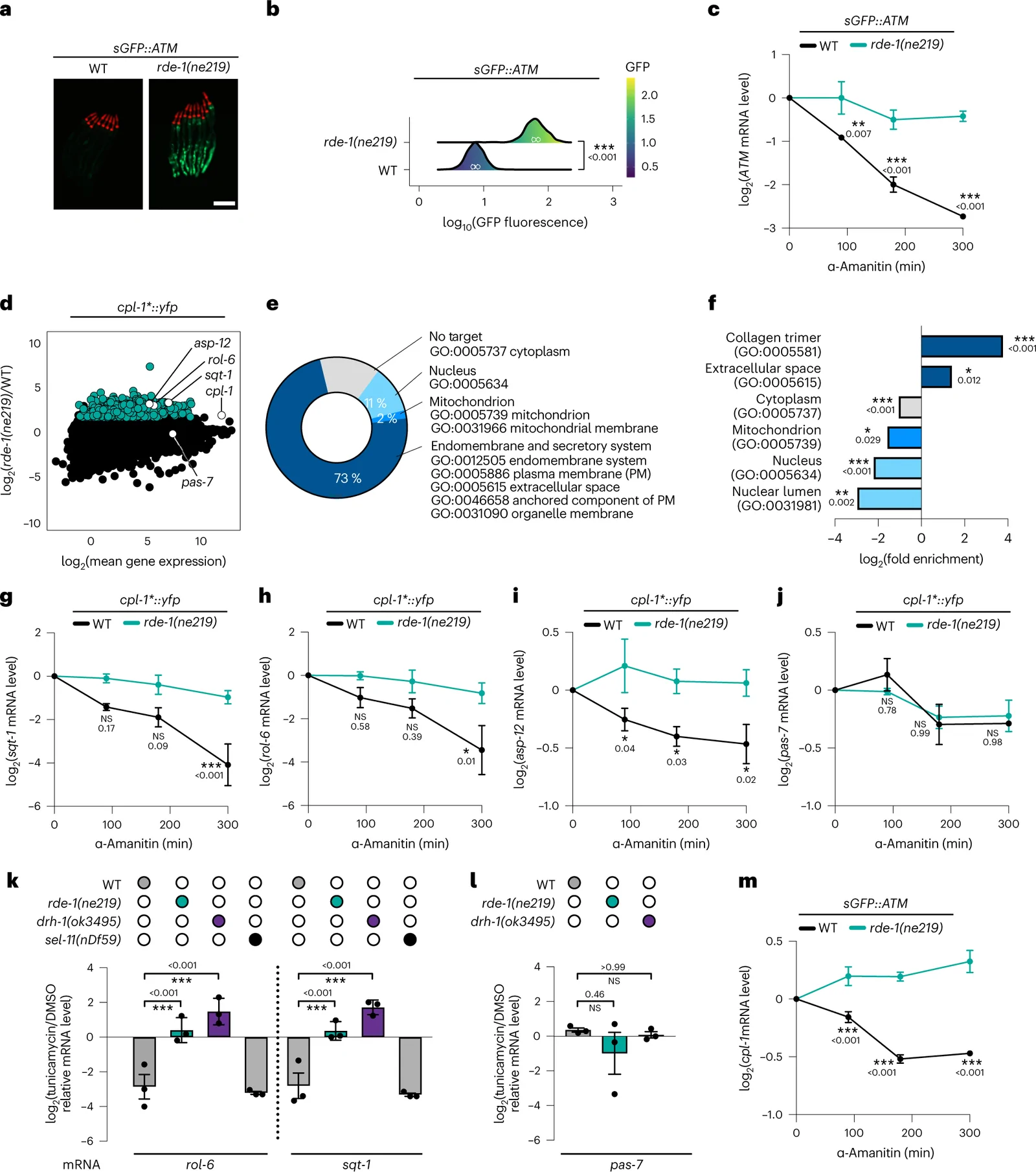
Endogenous mRNAs regulated by RDE-1. **a**, Fluorescence images of mutant worms for the exo-RNAi pathway expressing *ATM*. Pharyngeal expression of *Pmyo-2::mCherry* serves as transgenic marker. Scale bar, 200 µm. **b**, Quantification of GFP fluorescence intensity of *ATM* expressing worms together with *rde-1(ne219)* mutation. Measurements were carried out with the BioSorter using at least 200 animals per replicate, which were merged and displayed in ridgeline density plots. **c**, α-Amanitin chase assays for *ATM* mRNA from *rde-1(ne219)* mutants expressing *ATM*. mRNA measured by qRT–PCR. **d**, RNA sequencing results of cpl-1*-expressing worms bearing the *rde-1(ne219)* mutation or the WT allele. Cut-off for upregulated constructs: *P* ≤ 0.05. **e**, A total of 485 transcripts identified in **d** were subjected to BUSCA subcellular localization analysis. **f**, Subcellular component GO term enrichment for the genes identified in **d** and **e**. **g**–**j**, α-Amanitin chase assays for indicated endogenous mRNAs identified in **d** from *rde-1(ne219)* mutants and WT animals expressing cpl-1*. mRNA measured by qRT–PCR. **k**,**l**, Endogenous *rol-6*, *sqt-1* (**k**) and *pas-7* (**l**) mRNA level measured by qRT–PCR in vehicle (DMSO)- versus tunicamycin-treated WT worms. Data relative to 18S rRNA. **m**, α-Amanitin chase assays for endogenous *cpl-1* mRNA from *ATM* expressing animals with the *rde-1(ne219)* mutation or WT allele. mRNA measured by qRT–PCR. In c, g–j and m, data are relative to *cdc-42* and *pmp-3* mRNA. In **b**, data were analysed by pairwise *t*-test with Holmes multiple comparison test. In **k** and **l**, data were analysed by one-way ANOVA with Sidak’s multiple comparison test. In **c**, **g**–**j** and **m**, data were analysed by two-way ANOVA with Dunnett’s multiple comparison test. Values are depicted as mean ± s.e.m or, in **c** and **g**–**m**, as mean (white circles) ± standard deviation (s.d.) (white error bars). Data in **b** are generated from *n* = 3 independent experiments. *P < 0.05; **P < 0.01; ***P < 0.001; NS, *P* > 0.05. In **f**, raw *P* values were corrected for FDR. *FDR <0.05; **FDR <0.01; *** FDR <0.001; NS, FDR >0.05. Source numerical data are available in source data. Source data

**Extended Data Fig. 4:**
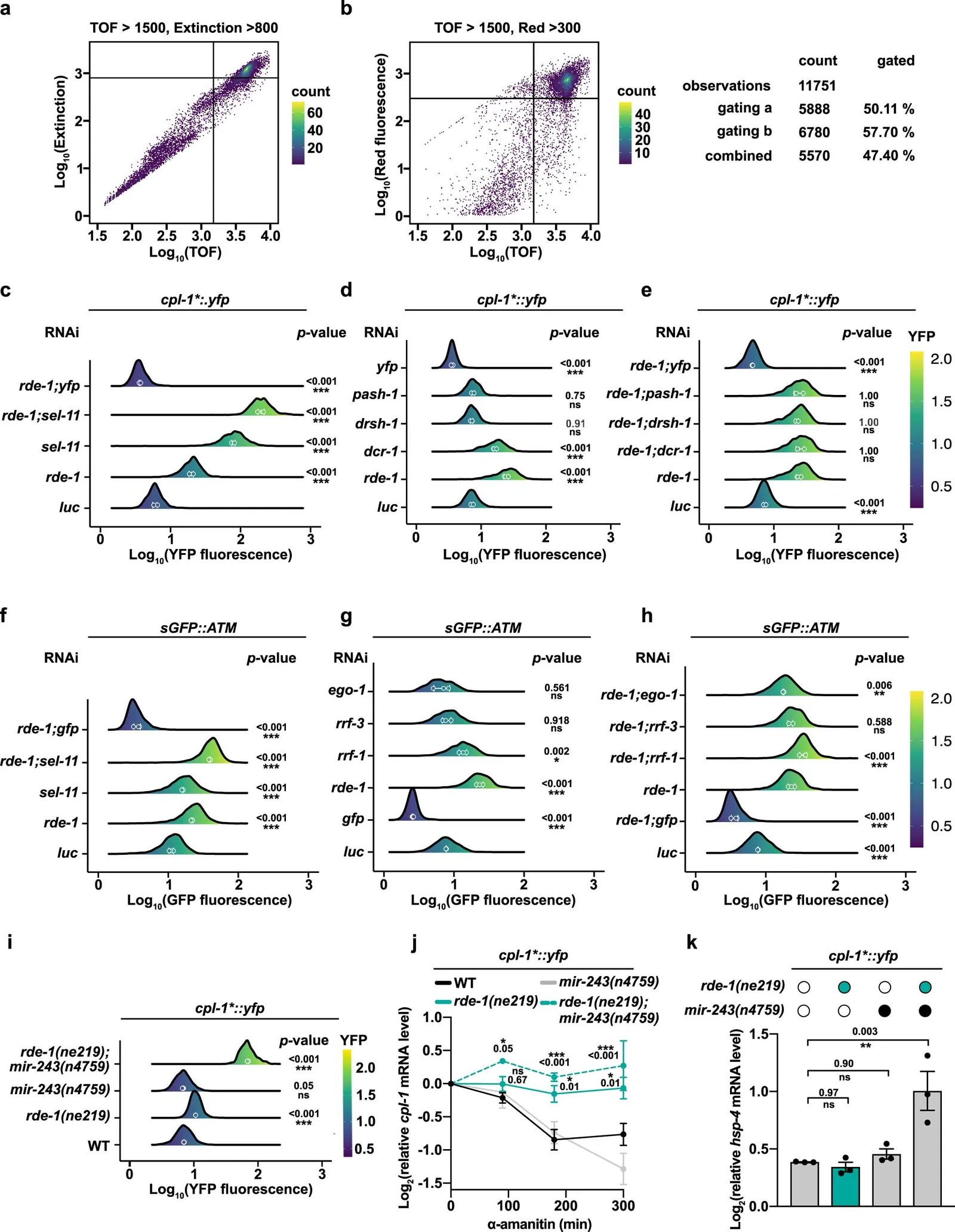
DCR-1 processed dsRNA species and RRF-1 are associated with ERAS. (**a, b**) 2d density hexbin plot (bins = 200) of all Biosorter® raw observations measured in Extended Data Fig. 4c, exemplifying the combined gating method of TOF, Extinction and Red fluorescence, which gates for day 1 adult worms co-expressing either the *ATM* or cpl-1* reporter with the pharyngeal *Pmyo-2::mCherry* (Red) transgenic marker (**c-e**) Quantification of YFP fluorescence intensity of cpl-1* expressing worms in RNAi treatments against ERAS and ERAD (c), miRNA maturation and dsRNA processing (d, e). *luc*: luciferase. (**f-h**) Quantification of GFP fluorescence intensity of *ATM* expressing worms in RNAi treatments against ERAS and ERAD (f) and secondary siRNA silencing (**g**). (**i**) Quantification of YFP fluorescence intensity of cpl-1* expressing worms with lof mutations for ERAS and/or *mir-243*. (**j**) Alpha-amanitin chase assays against cpl-1* mRNA in cpl-1* expressing worms with lof mutations for ERAS and/or *mir-243*, measured by qRT-PCR. Data relative to *cdc-42* and *pmp-3* mRNA. (**k**) *hsp-4* mRNA level measured by qRT-PCR in cpl-1* expressing worms with lof mutations for ERAS and/or *mir-243*. Data relative to *cdc-42* and *pmp-3* mRNA. [(c)-(i)] Measurements were carried out with the BioSorter^®^ using at least 200 animals per replicate, which were merged and displayed in ridgeline density plots. [(c)-(i)] were analyzed by pairwise t-test with Holmes multiple comparison test. (j) was analyzed by a two-way ANOVA with Sidak´s multiple comparison test. (k) was analyzed by one-way ANOVA with Dunnett’s multiple comparison test. Values are depicted as mean ± SEM [(j), (k)] or as mean (white circles) ± SD (white error bars) [(c)-(i)] generated from n = 3 independent experiments, *p < 0.05, **p < 0.01, ***p < 0.001, ns p > 0.05. WT: wild-type. Source numerical data are available in source data. Source data

To investigate whether other potential ERAD reporter substrates are also targeted by exo-RNAi components, we tested the *sGFP::ATM* (ATM) transgene, a signal peptide-GFP fused to human serpin that has previously been shown to reside in the ER^29^. Like the cpl-1* reporter, the ATM protein accumulates upon depletion of *rde-1* or *sel-11* (Fig. 4a,b and Extended Data Figs. 2c,d and 4a–c,f), which is enhanced upon double depletion of both factors (Extended Data Fig. 4a,d). In addition, the *ATM* transcript was strongly degraded by RDE-1 (Fig. 4c). Taken together, these data indicate continuous turnover of ATM protein and mRNA by SEL-11 and RDE-1, respectively.

Following the identification of exo-RNAi components as ER stress-dependent ERQC factors, we sought to characterize in vivo targets of the exo-RNAi machinery. To this end, we performed RNA sequencing and searched for upregulated mRNAs in *rde-1(ne219)* mutants in the context of exo-RNAi activation in cpl-1* expressing worms (Fig. 4d). We identified 485 mRNAs that were significantly upregulated and therefore represent potential exo-RNAi targets. By applying BUSCA analysis of subcellular localization^30^ we found that 73% of these candidates were associated with GO terms related to the endomembrane and secretory system, 11% to the nucleus and 2% to mitochondria (Fig. 4e and Supplementary Table 4). In addition, GO enrichment analysis for cellular components revealed significant positive enrichment for mRNAs encoding proteins with endomembrane involvement, such as collagen trimers and components of the extracellular space processed by the ER. In contrast, mRNAs encoding cytoplasmic, mitochondrial or nuclear factors showed significant negative enrichment (Fig. 4f and Supplementary Table 4).

As collagens are highly abundant throughout the animal kingdom and rely on ER import and proper ER homeostasis, we focused our analysis on the collagen trimer-encoding mRNAs *rol-6* and *sqt-1*. In addition, we tested *asp-12*, which encodes a lysosomal endopeptidase that, like the model substrate *cpl-1*, is highly expressed in the intestine. We found that all three exo-RNAi target candidates undergo RDE-1-dependent turnover in worms expressing the cpl-1* transgene (Fig. 4g–i). In contrast, mRNA of the proteasomal subunit *pas-7*, which was not upregulated in *rde-1(ne219)*, was not degraded by RDE-1 (Fig. 4d,j). Furthermore, we tested ER stress-dependent regulation of *rol-6* and *sqt-1* mRNAs by treatment with tunicamycin, which resulted in a strong decrease in *rol-6* and *sqt-1* transcript levels in WT and *sel-11* mutants but not in *rde-1* and *drh-1* mutants (Fig. 4k). In contrast, *pas-7* mRNA was also not regulated by RDE-1 in tunicamycin-treated worms (Fig. 4l). Taken together, our data suggest a previously unknown ER-associated RNA silencing mechanism regulated by ER folding capacity that contributes to ERQC. We termed this mechanism ERAS.

### DCR-1 is essential and RRF-1 conducive to ERAS

Argonaute proteins such as RDE-1 are controlled by DCR-1-processed double-stranded RNA (dsRNA) to regulate target RNAs (for example, mRNAs or viral RNAs). DCR-1, in turn, has a variety of substrates associated with endogenous mRNAs, including many collagens and, intriguingly, *cpl-1* (ref. ^31^). RDE-1 is heavily loaded with microRNAs (miRNA) and siRNA species, so the transcriptome may be examined for homology with DCR-1-processed dsRNA species^32^. Indeed, DCR-1 proved to be indispensable for ERAS, as depletion of DCR-1 phenotypically copies the loss of RDE-1 in terms of CPL-1* substrate stabilization (Extended Data Fig. 4d). Moreover, double depletion of *rde-1* and *dcr-1* did not further increase substrate levels (Extended Data Fig. 4e). Remarkably, we identified endogenous *cpl-1* mRNA as an RDE-1 target in animals expressing the ER-directed *ATM* substrate (Fig. 4m), demonstrating the natural potential of *C. elegans* to undergo an RNA silencing reaction against *cpl-1*. In contrast to DCR-1, the canonical miRNA biogenesis pathway mediated by DRSH-1 and PASH-1 was not required for the degradation of CPL-1* (Extended Data Fig. 4d,e). Furthermore, we tested whether RNA-dependent RNA polymerases *(rrf-1*, *rrf-3* and *ego-1*) contribute to substrate stabilization and confirmed the involvement of RRF-1, as knockdown of *rrf-1* leads to protein stabilization of the ATM substrate, albeit to a lesser extent than knockdown of *rde-1* (Extended Data Fig. 4g). These results recapitulate the effect of the *rrf-1(hh22)* mutant on CPL-1* (Fig. 1d,e). However, double depletion of *rrf-1* and *rde-1* showed an additive effect on protein substrate stabilization (Extended Data Fig. 4g,h), suggesting that RRF-1 and secondary siRNAs support RDE-1 even if they are not essential for its function in ERQC. We further investigated a possible role of *mir-243* in ER homeostasis, as *mir-243* was previously shown to physically interact with RDE-1, induce biogenesis of cognate siRNAs, and ultimately regulate its target transcript Y47H10A.5 (ref. ^32^). Accordingly, *mir-243(n4759)* LOF mutants were not shown to directly alter cpl-1* substrate stabilization at the protein or mRNA level (Extended Data Fig. 4i,j) because the cpl-1* transcript is not directly targeted by *mir-243*. However, *rde-1; mir-243* double mutants showed dramatically increased CPL-1* protein levels and strongly induced *hsp-4* mRNA expression (Extended Data Fig. 4i,k). GO enrichment analysis of published microarray data in *mir-243(n4759)* LOF mutants^32^ reveals an overrepresentation of the ‘unfolded protein response at the ER’ (false discovery rate (FDR) <0.01; Supplementary Table 4) in the upregulated gene group. Taken together, these data suggest that RDE-1-associated miRNAs such as *mir-243* are involved in the maintenance of ER homeostasis and confirm the role of ERAS in ERQC.

### AGO2 controls turnover of ER-associated mRNAs

**Fig. 5:**
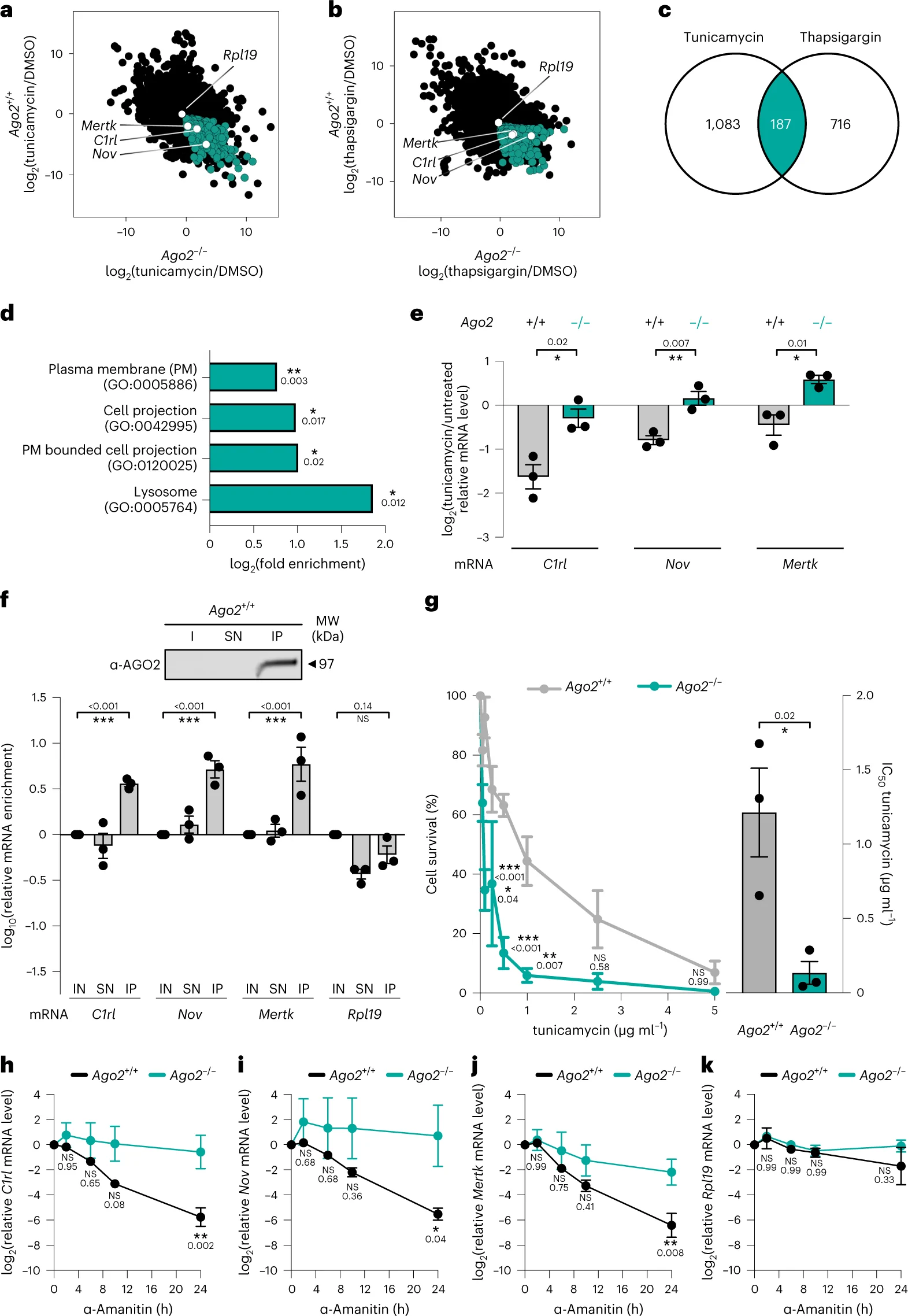
ERAS is conserved in mammalian cells. **a**,**b**, RNA sequencing results of tunicamycin/DMSO-treated (**a**) and thapsigargin/DMSO-treated (**b**) *Ago2*^*+/+*^ and *Ago2*^*−/−*^ MEF cells. Ago2-specific targets were selected by filtering (*P* ≤ 0.05) transcripts that were downregulated in *Ago2*^*+/+*^ and upregulated in *Ago2*^*−/−*^ cells (cyan). **c**, Venn diagram showing the number of transcripts regulated by AGO2 upon ER stress induction. Only transcripts that were identified in both ER stress conditions (**a** and **b**) were further investigated (cyan). **d**, Cellular component GO term enrichment of the genes identified in **a** and **b**. **e**, Expression levels of endogenous *Nov*, *Mertk* and *C1rl* mRNA in tunicamycin-treated MEFs measured by qRT–PCR. Data relative to 18S rRNA. **f**, UV CLIP of tunicamycin-treated MEF cells using AGO2 as bait and subsequent qRT–PCR of target mRNAs in the input (IN), supernatant (SN) and immunoprecipitation (IP) fractions. Data normalized to input fraction. Top shows western blot against AGO2 for the performed CLIP experiment. **g**, Dose-dependent cell survival after tunicamycin treatment calculated from the colony formation assay shown in Extended Data Fig. 5f. Bar graph showing the IC_50_ values for tunicamycin between *Ago2*^*+/+*^ and *Ago2*^*−/−*^ MEF cells. **h**–**k**, α-Amanitin chase assays for indicated endogenous mRNAs identified in **a** and **b** from *Ago2*^*+/+*^ and *Ago2*^*−/−*^ MEF cells pre-treated with tunicamycin. mRNA measured by qRT–PCR. Data relative to 18S and 28S rRNA. Data in **e** were analysed by unpaired two-tailed t-tests. Data in **f** were analysed by a two-way ANOVA with Dunnett’s multiple comparison test. The bar graph in **g** was analysed by paired two-tailed *t*-test. In **g**–**k**, data were analysed by two-way ANOVA with Sidak´s multiple comparison test. In **e**-**k** values are depicted as mean ± s.e.m. generated from *n* = 3 independent experiments, *P < 0.05; **P < 0.01; ***P < 0.001; NS, *P* > 0.05. In **d**, raw *P* values were corrected for FDR. *FDR <0.05; ** FDR <0.01; *** FDR <0.001; NS, FDR >0.05. Source numerical data and unprocessed blots are available in source data. Source data

**Extended Data Fig. 5:**
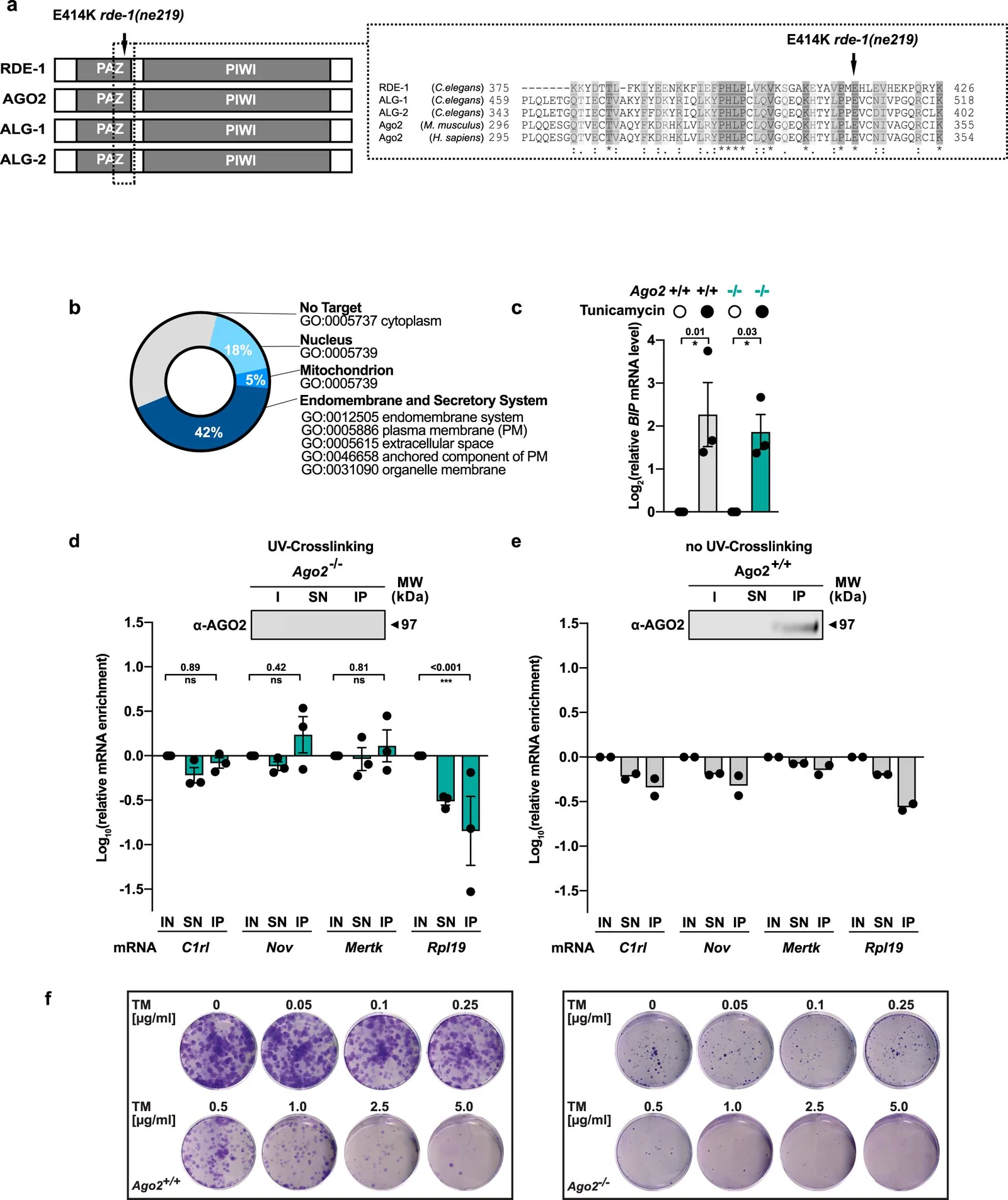
AGO2 binds to endogenous mRNAs and supports ER stress resistance. (**a**) Schematic illustration of PAZ-PIWI domain containing Argonaute proteins of *C. elegans* (RDE-1, ALG-1, ALG-2) and mammals (AGO2) showing the cross-species conservation of E414 in the PAZ domain, which is mutated to a lysine residue in the E414K mutation of the *rde-1*(*ne219)* allele. Aasterisk (*) indicates single conserved residues.:(colons) indicate conservation between groups of strongly similar properties, scoring >0.5 in the Gonnet PAM 250 matrix. Period (.) indicates conservation between groups of weakly similar properties, scoring = <0.5 in the Gonnet PAM 250 matrix. The alignment was performed by Clustal-Omega. (**b**) Genes identified in Fig. 5a-c were subjected to BUSCA subcellular localization analysis, showing a targeting of the obtained mRNAs to the endomembrane and secretory system. Results of the BUSCA analysis are summarized in Supplementary Table 4. **(c)** *BiP* mRNA level measured by qRT-PCR at the 0 h timepoint corresponding to the samples shown in Fig. 5h-k. Data relative to 18 S and 28 S rRNA. (**d, e**) Negative control experiments related to UV cross-linking immunoprecipitation (CLIP) experiments in Fig. 5f. CLIP of tunicamycin-treated *Ago2*^*−/−*^ (d) or in *Ago2*^*+/+*^ MEF cells but without UV cross-linking (e). AGO2 was used as bait with subsequent qRT-PCR against *C1rl*, *Nov*, and *Mertk* target mRNAs in the input (IN), supernatant (SN) and immunoprecipitation (IP) fractions. Data normalized to input fraction. Top panels show western blots against AGO2 for the performed CLIP experiment. (**f**) Images of tunicamycin (TM)-treated *Ago2*^*+/+*^ and *Ago*^*−/−*^ cell colonies stained with crystal violet. (c) was analyzed by unpaired two-tailed t-tests, [(d), (e)] were analyzed by a two-way ANOVA with Dunnett’s multiple comparison test. [(c), (d), (e)] Values are depicted as mean ± SEM generated from n = 3 [(c), (d)] or n = 2 (e) independent experiments, *p < 0.05, ***p < 0.001, ns p > 0.05. Source numerical data and unprocessed blots are available in source data. Source data

To investigate a possible role of ERAS in mammalian cells, we examined AGO2, which has high sequence similarity to RDE-1 in *C. elegans* and is functionally orthologous for siRNA-mediated RNA silencing in mammals^33^. AGO2 shares the same PAZ–PIWI domains and is conserved for the glutamate residue mutated in the *rde-1(ne219)* E414K allele (Extended Data Fig. 5a)^22,34^. We treated AGO2-deficient (*Ago2*^*−/−*^) mouse embryonic fibroblasts (MEFs)^20^ with the ER stressors tunicamycin or thapsigargin. To identify ERAS targets, we filtered for mRNAs that were upregulated in *Ago2*^*−/−*^ cells and downregulated in wild-type (*Ago2*^*+/+*^) control cells for both ER stressors (Fig. 5a,b). BUSCA analysis of subcellular localization revealed that of the 187 candidates (Fig. 5c) 42% had GO terms associated with the endomembrane and secretory system, 18% with mitochondria and 5% with the nucleus (Extended Data Fig. 5b)^30^. GO term analysis showed significant enrichment of mRNAs associated with cell projection, plasma membrane (PM), PM bounded cell projection and lysosomes (Fig. 5d).

We selected three mRNAs from candidate genes representing ER luminal (*C1rl*), PM (*Mertk*) and secreted (*Nov*) proteins and confirmed AGO2-dependent regulation for all three mRNAs upon tunicamycin-induced ER stress (Fig. 5e and Extended Data Fig. 5c). UV-crosslinking immunoprecipitation (CLIP) for AGO2 combined with qRT–PCR revealed direct protein–RNA binding between AGO2 and the mRNAs *C1rl*, *Nov* and *Mertk* (Fig. 5f). We were able to enrich the *C1rl*, *Nov* and *Mertk* transcripts in *Ago2*^*+/+*^ cells but not in *Ago2*^*−/−*^ cells or negative UV-crosslinking controls, demonstrating the specificity of the assay (Fig. 5f and Extended Data Fig. 5d,e). In contrast, the housekeeping mRNA *Rpl19*, which encodes the 60S ribosomal protein L19 and whose expression did not change significantly in the RNA-sequencing assay (Fig. 5a,b), was not enriched in AGO2 CLIP-qRT-PCR (Fig. 5f). We also tested the ability of single *Ago2*^*−/−*^ cells to form a colony after tunicamycin treatment. Compared with *Ago2*^*+/+*^ controls, *Ago2*^*−/−*^ cells showed reduced survival with increasing tunicamycin dose, suggesting a cytoprotective ERQC function of ERAS in mammals (Fig. 5g, and Extended Data Fig. 5f). Strikingly, the endogenous mRNAs bound by AGO2 in the CLIP qRT–PCR assay (Fig. 5f) were indeed degraded in an AGO2-dependent manner upon ER stress exerted by tunicamycin pre-treatment (Fig. 5h–j), whereas *Rpl19* mRNA showed no AGO2-mediated turnover (Fig. 5k). Thus, downregulation of ER-associated RNAs by the Argonaute proteins RDE-1/AGO2 upon ER stress is conserved from worms to mammals.

### ERAS and ERAD work together to maintain ER homeostasis

**Fig. 6:**
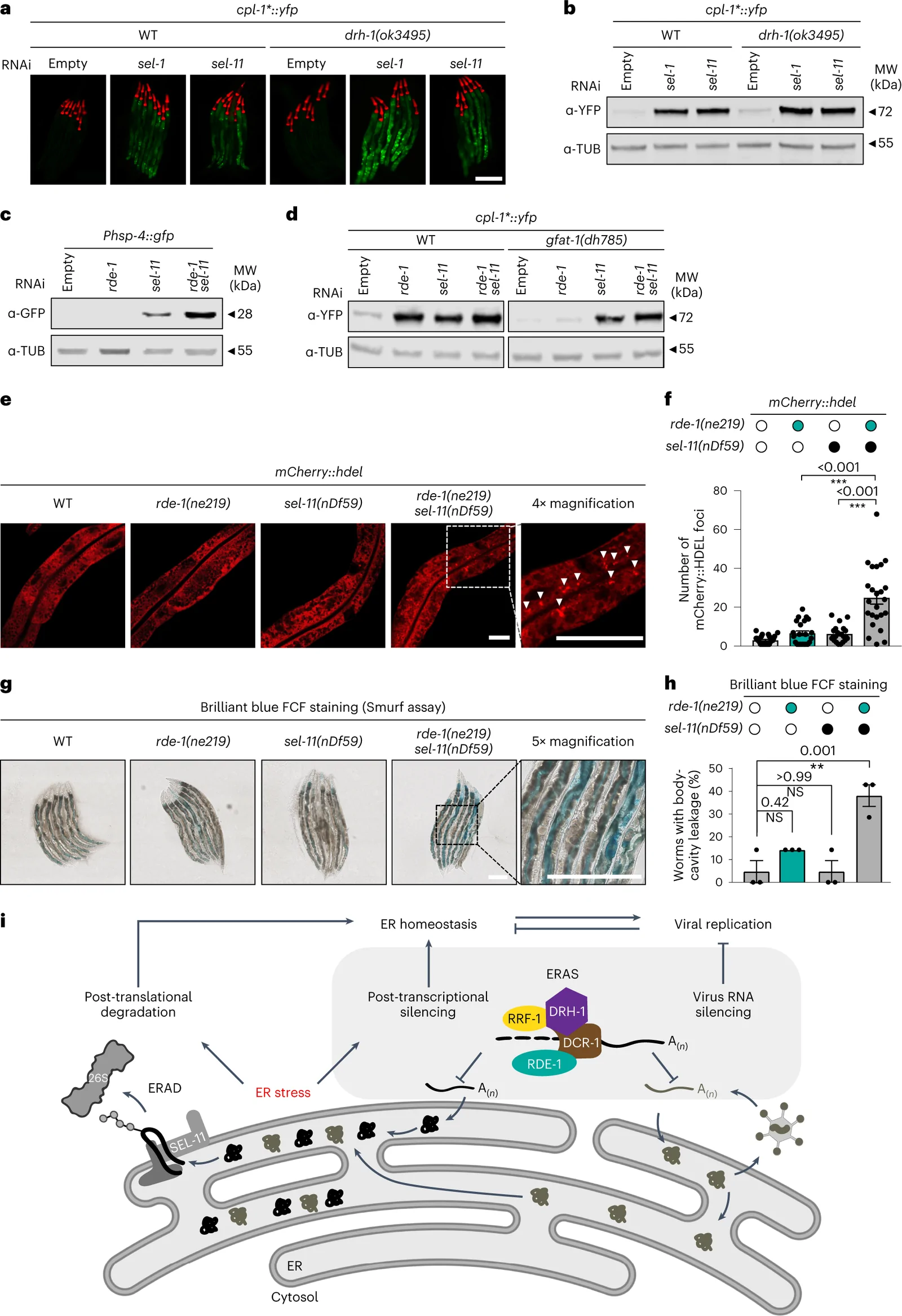
ERAS and ERAD execute complementary functions in ERQC. Fluorescence images of worms expressing cpl-1*. Pharyngeal expression of *Pmyo-2::mCherry* serves as transgenic marker. Scale bar, 200 µm. Images were taken with lower exposure time to prevent YFP overexposure for animals with defects in both, the ERAS and ERAD pathway. **b**, Western blot of worm lysates corresponding to the samples in **a**, detecting CPL-1* (YFP) and tubulin (TUB). **c**, Western blot of worm lysates with the indicated RNAi treatment, detecting GFP (GFP) and tubulin (TUB). **d**, Western blot of worm lysates from worms depleted for ERAS and/or ERAD components expressing cpl-1* in the WT or *gfat-1* GOF background detecting CPL-1* (YFP) and tubulin (TUB). **e**, Confocal microscopy images showing worms expressing the ER marker *mCherry::hdel* (red). Focal accumulation of mCherry highlighted by arrow heads. Scale bars, 20 µm. **f**, Quantification of mCherry-HDEL foci shown in **e**, *n* = 28, 22, 21 and 24 animals (left to right). **g**, Intestinal barrier (Smurf) assay. Representative images of day 5 adult worms soaked in blue food dye (Brilliant Blue FCF). Blue dye leaking from the intestinal lumen into the body cavity gives rise to the Smurf phenotype. Scale bars, 200 µm. **h**, Quantification of body-cavity leakage in animals shown in **g**, *n* = 7 individual animals/*n* = 3 replicates. In **f** and **h**, data were analysed by one-way ANOVA with Bonferroni’s multiple comparison test and values are depicted as mean ± s.e.m. generated from *n* = 3 independent experiments, **P < 0.01; ***P < 0.001; NS, *P* > 0.05. **i**, The ERAD machinery detects misfolded proteins and retro-translocates them into the cytosol for polyubiquitylation by SEL-11 and proteasomal turnover. The ERAS machinery mediates silencing of ER-associated mRNAs and viral RNA. ERAD and ERAS are required upon ER stress caused by misfolded proteins and virus replication and their complementary functions contribute to maintain ER homeostasis and organismal functionality. Source numerical data and unprocessed blots are available in source data. Source data

**Extended Data Fig. 6:**
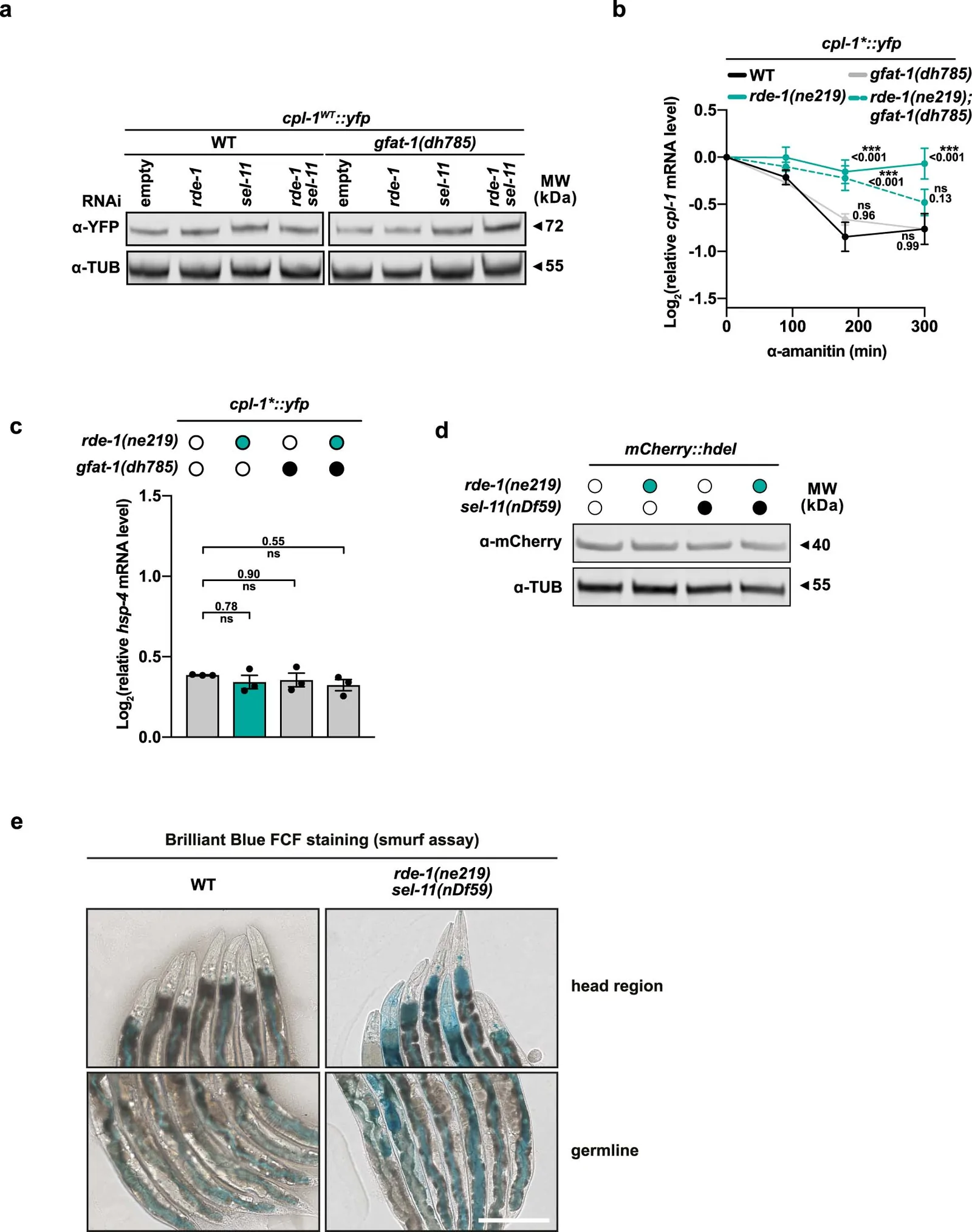
ERAS and ERAD maintain ER quality control and intestinal integrity. (**a**) Western blot of worm lysates from worms depleted for ERAS and/or ERAD components expressing *cpl-1*^*WT*^ in the WT (wild-type) or *gfat-1* gain-of-function background detecting CPL-1^WT^ (YFP) and tubulin (TUB). (**b**) Alpha-amanitin chase assays against cpl-1* mRNA from ERAS mutants and/or animals carrying a *gfat-1* gain-of-function mutation expressing cpl-1*, measured by qRT-PCR. (**c**) *hsp-4* mRNA level measured by qRT-PCR at the 0 h time point corresponding to the samples shown in (b). (**d**) Western blot of worm lysates corresponding to the data shown in Fig. 6e detecting mCherry::HDEL (mCherry) and tubulin (TUB). (**e**) Images showing the head and germline region of worms with the indicated genotypes after soaking in blue food dye (Brilliant Blue FCF). Blue dye leaking from the intestinal lumen into the body cavity gives rise to the Smurf phenotype. Scale bar: 200 µm. [(b), (c)] Data relative to *cdc-42* and *pmp-3* mRNA. (b) was analyzed by two-way ANOVA with Dunnett’s multiple comparison test, (c) was analyzed by one-way ANOVA with Sidak’s multiple comparison test. [(b), (c)] Values are depicted as mean ± SEM generated from n = 3 individuals, ***p < 0.001, ns p > 0.05. Source numerical data and unprocessed blots are available in source data. Source data

To understand the regulatory relationship between post-transcriptional and post-translational ERQC, we combined ERAS and ERAD mutants and tracked changes in CPL-1* protein levels. ERAS-defective *drh-1* mutants combined with *sel-1* or *sel-11* depletion showed enhanced CPL-1* accumulation compared with *sel-1* and *sel-11* single mutants (Fig. 6a,b), recapitulating the effects of double depletion of *rde-1* and *sel-11* on CPL-1* and ATM protein (Extended Data Fig. 4a,d). Whereas knockdown of *sel-11* slightly induced expression of the ER stress reporter *Phsp-4::gfp*, this was significantly increased by the additional depletion of *rde-1*, highlighting the role of ERAS in ERQC, especially when ERAD is impaired (Fig. 6c). To distinguish the mechanism of RNA silencing from that of protein degradation in ERAS and ERAD, respectively, we used glutamine fructose 6-phosphate aminotransferase (*gfat-1*) gain-of-function (GOF) mutants, which exhibit enhanced glycosylation and ER protein folding and thus enhanced glycan-dependent ERAD^27^. We found that *gfat-1* (GOF) mutants were able to fully suppress ERAS-mediated but not ERAD-mediated CPL-1* substrate accumulation, confirming a role for GFAT-1 in enhancing ERAD (Fig. 6d). In contrast, simultaneous inhibition of ERAS and ERAD in *gfat-1* (GOF) mutants showed an additive effect, indicating that *gfat-1* (GOF) was no longer able to suppress ERAS-mediated CPL-1* substrate accumulation in the presence of dysfunctional ERAD (Fig. 6d). Moreover, *gfat-1* (GOF) had no effect on the correctly folded and ERAD-independent CPL-1^WT^ protein (Extended Data Fig. 6a). Finally, we found that *gfat-1* (GOF) had no effect on ERAS activity at the mRNA level of the cpl-1* substrate (Extended Data Fig. 6b), which is in line with the observation that baseline *hsp-4* expression remains constant in *gfat-1* (GOF) mutants^27^ (Extended Data Fig. 6c). Overall, our data show that ERAS and ERAD work together to maintain ER homeostasis by reducing the protein folding load of the ER.

To investigate the physiological consequences of simultaneous impairment of ERAS and ERAD, we monitored ER protein folding capacity and intestinal integrity, representing changes at the cellular and organismal level. The mCherry-HDEL fusion protein, which is targeted to the ER lumen by a SEL-1 signal sequence^35,36^, requires functional protein quality control in the ER for correct folding. Confocal imaging revealed focal accumulations of mCherry-HDEL protein with concomitant impairment of ERAS and ERAD (Fig. 6e,f). To exclude the possibility that the observed effects were due to altered transgene expression in the different mutant backgrounds, we monitored mCherry-HDEL protein level, which remained unchanged in ERAS and ERAD mutants (Extended Data Fig. 6d). These findings further underline a cooperation between ERAS and ERAD in maintaining ER homeostasis.

ER stress has been associated with loss of intestinal homeostasis, including the development of inflammatory bowel disease and loss of epithelial barrier integrity^37,38^. To assess intestinal integrity of *rde-1 sel-11* double mutants, we tested intestinal barrier function in *C. elegans*^39^. Animals were fed a blue food dye, and leakage of the dye from the intestine into the body cavity (Smurf phenotype) was observed microscopically. While single mutants were able to retain the dye in the intestine, *rde-1 sel-11* double mutants showed severely compromised intestinal barrier integrity (Fig. 6g,h and Extended Data Fig. 6e). Taken together, our results identify ERAS as an RNA surveillance mechanism that regulates the turnover of ER-associated RNAs and viral RNAs to maintain ER homeostasis and organism functionality (Fig. 6i).

## Discussion

ER homeostasis is disrupted by the accumulation of misfolded luminal proteins, which are generally degraded via ERAD^8^. Here we identified a conserved post-transcriptional pathway with a complementary function to ERAD in ERQC, which we termed ERAS. ERAS consists of the exo-RNAi machinery in worms and focuses on RDE-1/AGO2, a member of the Argonaute protein family that exists in plants, *Drosophila* and vertebrates^22^. DCR-1, DRH-1 and RRF-1 are also involved in the turnover of ER-targeted mRNAs during ER stress (Figs. 1d,e and 2c,d and Extended Data Figs. 2a and 4e–h). Here we show that the ERAS machinery targets a variety of endogenous mRNAs encoding proteins of the endomembrane and secretion system (Fig. 4e and Extended Data Fig. 5b). Although ERAS and ERAD work together to maintain ER protein homeostasis, their mechanisms of action are distinct. ERAS works through the cellular RNA silencing machinery for mRNA degradation, whereas ERAD uses the ubiquitin/proteasome system for protein degradation (Figs. 2a–d and 6d and Extended Data Figs. 4c,f,g–i and 6a,b). We show that ERAS is triggered by ER stress induced by expression of the ERAD substrates CPL-1* and ATM, treatment with tunicamycin/thapsigargin, or viral infection (Figs. 2c,d,g,i, 3a–c, 4c,k and 5 and Extended Data Figs. 2g and 3c). Similarly, defects in the ERAD or UPR^ER^ pathways triggered stress that significantly reduced the levels of ERAS-targeted RNAs, as shown in *sel-1*, *sel-11*, *atf-6*, *pek-1* and *ire-1* LOF mutants (Figs. 2i and 3d,e and Extended Data Fig. 3a), indicating the interdependence of ERAD, UPR^ER^ and ERAS in mediating ERQC and viral defence. The cooperation of ERAS and ERAD is crucial, as shown by the enhanced upregulation of UPR^ER^, reduced protein quality control and impaired intestinal integrity when both ERAD and ERAS are defective (Fig. 6c,e–h). In addition to anti-viral defence, the exo-RNAi pathway may also play a role in silencing extrachromosomal transgenes, which has not yet been fully elucidated^40,41^. Our data rule out ERAS being affected by indirect transgene silencing effects, on the basis of its causal relationship with ER homeostasis (Figs. 2g, 3a–d, 4a–c, 5a–g and 6d and Extended Data Figs. 3a, 4c,f and 6a,b), constitutive degradation of cpl-1* but only facultative turnover of *cpl-1*^*WT*^ (Fig. 3a,b and Extended Data Fig. 3a,b), the identification of endogenous ERAS-targets in worms and MEFs (Figs. 4g–m and 5a–f,h–k), and finally the specific regulation of cpl-1*, cpl-1^*WT*^ and *ATM* compared with other transgenes used in this study (Figs. 3c,d, and 6c,d and Extended Data Fig. 6a,c). RDE-1 mediates RNA silencing by binding to the 3′ end of Dicer-processed dsRNAs via its conserved PAZ domain^22^. As regulation of ER-targeted RNAs is abolished in *rde-1(ne219)* worms with a single point mutation in the PAZ domain (Figs. 2c,d,g, 3c and 4c,d,g–m and Extended Data Figs. 2b, 3b and 4c,f), we propose a comparable mechanistic function of RDE-1 in ERAS. This conclusion is supported by the fact that AGO2 specifically binds and degrades mRNA targets that accumulate in *Ago2*^*−/−*^ cells under ER stress conditions (Fig. 5f,h–k and Extended Data Fig. 5d,e). However, the precise mechanisms by which ER stress triggers RDE-1/AGO2-mediated mRNA silencing remain unclear. Recent evidence suggests that the ER membrane is a central hub for RNA silencing. Membrane fractionation and immunostaining experiments have shown that siRNA-loaded AGO2 binds to the cytosolic side of the rough ER membrane along with Dicer and that induction of ER stress by thapsigargin treatment repels AGO2-bound mRNAs from the ER^42,43^. Given that *Ago2*^*−/−*^ cells are sensitive to tunicamycin treatment (Fig. 5g and Extended Data Fig. 5f), these data suggest a conserved role for *Ago2*-mediated RNA silencing in ER homeostasis. Interestingly, several UPR^ER^-inducible miRNAs were found to regulate the cellular survival response by directly targeting components of the UPR^ER^ such as CHOP, Xbp-1 or Caspase-2 (ref. ^44^). The UPR^ER^-inducible miRNA *miR-708* regulates rhodopsin, which is produced in large amounts in the ER and is an important component of the developing retina. Through *Ago2*-dependent downregulation of rhodopsin upon ER stress, *miR-708* prevents the accumulation of misfolded rhodopsin in the ER, highlighting the physiological importance of ERAS^44,45^. The description of an miRNA-directed siRNA biogenesis pathway, as demonstrated for *mir-243* (ref. ^32^), together with our results, suggests a highly adaptive RNA regulatory capacity of ERAS and may provide an exciting avenue for further studies to elucidate tissue-specific small RNA regulatory circuits that regulate ERQC. Conceptually, any DCR-1 processed small RNA species could be involved in ERAS. A recent publication also found that AGOs localized at the ER promote ubiquitylation of nascent polypeptide chains by the E3 ligase Ltn1 (ref. ^46^). This raises the intriguing idea that AGO proteins represent interaction nodes for coupling post-transcriptional gene silencing and protein quality control at the ER. However, our results suggest that ERAS does not specifically degrade the mRNA of an unstable protein species, but that multiple ER stress-inducing factors such as cpl-1*, *ATM*, tunicamycin, thapsigargin, *sel-11* LOF and viral infection act on ERAS to mediate ERQC, independent of the protein fold status of the gene product (Figs. 2g–i, 3c,d, 4g–m and 5 and Extended Data Fig. 3).

Many viruses use the endomembrane system for cell entry, replication and assembly. However, the causal relationship between anti-viral defence by RNA silencing and ER homeostasis has been underestimated. We cannot completely exclude the possibility that the decreased viral RNA level in response to ER stress is due to decreased viral replication rather than ERAS-mediated RNA degradation (Fig. 2g–i). However, the reduction of viral RNAs upon pre-treatment with ER stress is conserved in mammals^47,48^; for example, non-cytotoxic pre-treatment with thapsigargin effectively protected mice from otherwise lethal doses of influenza A^48^, and cells pre-treated with thapsigargin showed a dramatic reduction in viral RNA levels in cells infected with severe acute respiratory syndrome coronavirus 2 (ref. ^47^). These two RNA viruses use the ER and endomembrane system for their replication^49–51^. From an evolutionary perspective, we speculate that ERAS has co-opted an ancient anti-viral pathway into a general RNA surveillance mechanism that regulates ERQC. Our results may therefore integrate these phenomena into a coherent model of how ERAS mediates ERQC and viral defence (Fig. 6i).

## Methods

### *C. elegans* maintenance and transgenic lines

Nematodes were grown at 20 °C (unless stated otherwise) on nematode growth medium (NGM) plates seeded with the bacterial *Escherichia coli* strain OP50 as a food source according to standard protocols and methods^52,53^. The N2 Bristol strain served as wild-type. If not stated otherwise, all experiments were performed at day 1 of adulthood, and the examined worms were all hermaphrodites. Integration of the extrachromosomal arrays of the *cpl-1*^*WT*^*::yfp* and cpl-1*::yfp strains obtained from Gary Silverman^13^ was conducted by UV-induced DNA double-strand breaks. Synchronized L4 larvae were irradiated with 30 Gy UV light and cultured for 2 h at room temperature (RT). Eight worms per plate were picked onto 20 NGM plates (100 mm) and were grown until the F2 generation (approximately 5 days at 20 °C). Five-hundred worms of the obtained progeny were isolated on NGM plates (35 mm). The F3 generation was screened for 100% penetrance for the transgenic markers. If not stated otherwise, ER stress in worms was induced by treatment with 5 µg ml^−1^ tunicamycin (Sigma-Aldrich) for 8 h. Dimethyl sulfoxide (DMSO) (v/v) served as solvent control. All worm and bacterial strains that were used in this study are listed in Supplementary Table 1.

### Mammalian cell lines

*Ago2*^*−/−*^ MEF and *Ago2*^*+/+*^ MEF cells were a kind gift from Gunter Meister (University of Regensburg). Cells were grown at 37 °C and 5% CO_2_ in Dulbecco’s modified Eagle medium GlutaMAX (Gibco) supplemented with 10% foetal calf serum and 1% penicillin–streptomycin. Cells were passaged every 3–4 days (at ~80% confluency) by trypsinization with 0.5% trypsin–EDTA. If not stated otherwise, ER stress in cells was induced by treatment with 3 µg ml^−1^ tunicamycin (Sigma-Aldrich) for 4 h. DMSO (v/v) served as solvent control.

### Western blotting

For the preparation of whole worm lysates, animals were collected in M9 buffer and washed twice. Worm pellets were stored at −80 °C or used immediately for downstream applications. Worm pellets were resuspended in cooled RIPA buffer (150 mM NaCl, 1% Triton X-100, 0.5% sodium deoxycholate, 0.1% sodium dodecyl sulfate (SDS) and 50 mM Tris (pH 8.0)). Subsequently, samples were boiled at 95 °C for 5 min, sonicated (30 s at 60% amplitude, Bandelin Sonoplus with MS 1.5 sonotrode) and boiled again at 95°C for 5 min. Lysates were cleared by centrifugation (5 min, 21,000 relative centrifugal force (RCF), 4°C) and the protein concentration was determined with the Pierce BCA Protein Assay Kit (Thermo Fisher Scientific). Samples were then mixed with 2× SDS loading buffer. For analysis and size separation of proteins, SDS gel electrophoresis was performed (PAGE)^54^. NuPAGE 4–12% Bis-Tris SDS gels were used with the respective NuPAGE MES SDS running buffer (Thermo Fisher Scientific; settings according to the manufacturer’s instructions). For the transfer of proteins to a nitrocellulose membrane (Amersham, Protran 0.2 µm), a semi-dry blotting system (Bio-Rad, Trans-Blot Turbo) was used. Standard transfer was performed with 25 V (constant) for 30 min using NuPAGE transfer buffer, supplemented with 10% methanol. After blotting, the membrane was directly incubated with the respective primary antibody, diluted in 1× Roti-Block (Carl Roth) overnight at 4 °C while constantly shaking. For detection of AGO2, the T22 Mighty Small wet transfer system (Hoefer; 11 V, overnight at 4 °C) with wet transfer buffer (25 mM Tris, 192 mM glycine, 0.01% SDS and 20% methanol) was used. The membrane was blocked with 5% milk powder (Carl Roth) in phosphate-buffered saline (PBS)–Tween (137 mM KCl, 8.1 mM Na_2_HPO_4_, 1.5 mM KH_2_PO_4_ and 0.1% (v/v) Tween-20, pH 7.4) for 1 h at RT, before incubation with primary antibodies (in 5% milk powder (Carl Roth) in PBS–Tween) overnight at 4 °C. Subsequently, the membrane was washed three times with PBS–Tween for approximately 10 min. The membrane was further incubated with the respective fluorescently labelled secondary antibody (LI-COR Biosciences) for 1 h at RT and subsequently washed again three times with PBS–Tween for approximately 10 min. Final visualization was performed using the Odyssey scanner (LI-COR Biosciences) and respective software (Image Studio Software v4.0 or v5.0, LI-COR Biosciences). Western blots were quantified using the Image Studio Software v4.0 or 5.0 (LI-COR Biosciences). The antibodies that were used in this study were as follows (see also Supplementary Table 2): mouse monoclonal anti-α-tubulin (B-5-1-2) (Sigma, catalogue number T6074, 1:5,000); mouse monoclonal anti Living Colors (JL-8, anti-GFP) (Clontech Laboratories, catalogue number 632380, 1:5,000); mouse monoclonal anti Living Colors, DsRed (anti-mCherry) (Clontech Laboratories, catalogue number 632393, 1:5,000); anti-CDC-48.1 (Hoppe-lab/Biogenes Berlin, custom antibody, 1:50,000); anti-SEL-1, (Sommer-lab, Berlin, custom antibody, 1:8,000); anti-Ago2 monoclonal (C34C6) (Cell Signaling catalogue number 2897, 1:10,000; anti-α (orsay virus) (Wang-lab, Washington, custom antibody, 1:2,000); IRDye 800CW donkey anti-mouse IgG (H + L) (LI-COR Biosciences, catalogue number 926-32212, 1:10,000); IRDye 800CW donkey anti-rabbit IgG (H + L) (LI-COR Biosciences, catalogue number 926-32213, 1:10,000); IRDye 680 donkey anti-mouse IgG (H + L) (LI-COR Biosciences, catalogue number 962-32222, 1:10,000); IRDye 680 donkey anti-rabbit IgG (H + L) (LI-COR Biosciences, catalogue number 962-32223, 1:10,000).

### EMS mutagenesis

A total of 10,000 synchronized L4 larvae (*Pnhx-2::cpl-1*^*Y32A,W35A*^*::yfp*) were washed three times with M9 buffer (20 mM KH_2_PO_4_, 40 mM Na_2_HPO_4_, 80 mM NaCl and 1 mM MgSO_4_) and subsequently incubated with 50 mM EMS while shaking for 4 h at RT. Further, the worms were washed three times with M9 buffer and incubated for 24 h at 20 °C on NGM plates. The microscopy-based selection process for elevated CPL-1* level was performed at the F_2_ generation using the Leica M80 fluorescent microscope. Selected worms were outcrossed twice with wild-type N2.

### CHX chase assay

Cycloheximide (CHX) interferes with the ribosomal translocation step during protein synthesis and thereby blocks eukaryotic protein translation, which makes it a valuable tool to monitor time-dependent protein degradation rates. NGM plates (90 mm) were coated with 200 µl CHX (50 mg ml^−1^ in ethanol, Sigma-Aldrich) or with 200 µl ethanol and incubated for 30 min at RT. Subsequently the plates were seeded with 1,000 µl *E. coli* OP50 and incubated over night at 37 °C. A total of 400–600 worms each were distributed on each plate and incubated at 20 °C for 0 h, 1 h, 3 h, 6 h and 9 h, respectively. After the incubation time, worms were collected and analysed via western blot and BioSorter. For each timepoint, the treated/untreated (CHX/EtOH) ratio was calculated and normalized by dividing by the 1 h timepoint ratio.

### BioSorter quantification of fluorescence

The BioSorter (Union Biometrica) with the FlowPilot-Pro software is a large-particle flow cytometer that allows for the quantification of fluorescence in three different channels ‘Red’, ‘Yellow’, ‘Green’ and parameters such as time-of-flight (TOF) and extinction. TOF, Red and extinction were used for gating of observations, which resembled day 1 adult worms expressing either cpl-1* or *ATM* exhibiting pharyngeal expression of *Pmyo-2::mCherry* as transgenic marker (Figs. 1b,d and 4a). Unless stated otherwise, after gating (TOF >1,500, Red >300 and extinction >800), the fluorescence values (YFP yellow or GFP green) of at least 200 gated worms from three independent biological replicates were used to calculate mean ± standard deviation (s.d.) values and plot density ridgeline plots (ggplot2 v.3.6.2 and ggridges v.3.6.2). Experiments with smaller *sel-11(nDf59)* mutant worms (Fig. 2b) were gated with (TOF >1200, Red >300 and extinction >600). A graphical illustration of the gating method is presented with a 2d density hexbin plot (hexbin v.1.28.2) in Extended Data Fig. 4a,b.

### RNA extraction

Isolation of total RNA was performed using TRIzol (Invitrogen). Age-synchronized worms were collected and washed twice with M9 buffer. Finally, 1 ml of TRIzol was added and worms were frozen at −80 °C for at least 1 h. Subsequently, worms were thawed at 37 °C. Disruption of worms and RNA release was achieved by using silica beads and the Precellys 24-Dual cell homogenizer (Peq-Lab) twice for 20 s at 5,000 rpm. Samples were incubated for 15 min at RT. For RNA extraction from mammalian cells, the growth medium was removed and cells were washed once with PBS. Subsequently, cells were scratched from the culture dish, collected in PBS and pelleted by centrifugation (5 min, 500 RCF, RT). The supernatant was removed, the pellet resuspended in 1 ml TRIzol and samples frozen at −80 °C for at least 1 h. Next, 100 µl 1-bromo-3-chloropropane was added to the cell culture or *C. elegans* samples and centrifuged for 15 min at 8,000 RCF at 4 °C to separate aqueous and organic phase. The aqueous phase was used to isolate total RNA with the RNeasy Mini Kit (Qiagen) following the manufacturer’s instructions or by isopropanol–glycogen precipitation. In brief, 1 µl glycogen (10 mg ml^−1^) and 500 µl of isopropanol was added. The samples were subsequently incubated for 1 h at −80 °C. The samples were further centrifuged for 45 min at 16,000 RCF at 4 °C. The supernatant was removed, and the pellet was washed with 70% ethanol. Then the RNA was air dried and resuspended in nuclease-free water. Quality and concentration of the isolated RNA was measured using the NanoDrop 8000 spectrophotometer (Thermo Fisher Scientific).

### cDNA synthesis and qRT–PCR

A total of 1,000–2,000 ng of total RNA was used for cDNA synthesis with the High-Capacity cDNA Reverse Transcription kit (Applied Biosystems) following the manufacturer’s instructions. Measurement of cDNA level was performed with Luna Universal qPCR Master Mix (New England Biolabs) and the Bio-Rad CFX96 Real-Time PCR Detection System. Three technical replicates were analysed per sample. As reference for normalization, 18S ribosomal RNA (rRNA) *cdc-42* and *pmp-3* mRNA (*C. elegans*) or *Rpl19* mRNA, 18S rRNA and 28S rRNA (MEF cells) were used. All oligonucleotides that were used in this study are listed in Supplementary Table 3.

### α-Amanitin chase assay

Worms of the respective genotypes were L1 synchronized on NGM plates by filtering and exposed at day 1 of adulthood to α-Amanitin (50 μg ml^−1^, Sigma-Aldrich) in liquid culture (M9 with OP50 bacteria as food source (*t* = 0 h). For monitoring mRNA degradation for shorter time periods (up to 300 min), 0.1% Triton X-100 was added to the reaction to improve α-Amanitin uptake. Samples were collected in TRIzol after the respective timepoints of the transcriptional block, purified by RNA extraction and cDNA synthesis, and analysed by qRT–PCR for the constructs of interest as described above. For α-Amanitin chase assays in mammalian cells, 160,000 MEF cells were seeded in six-well plates. Two days later, ER stress was induced with 3 µg ml^−1^ tunicamycin (Sigma-Aldrich) for 4 h. Adding only DMSO (v/v) served as solvent control. Next, tunicamycin was removed, α-Amanitin (35 µg ml^−1^) was added and cells were collected at 0 h, 2 h, 6 h, 10 h and 24 h after treatment in TRIzol, followed by RNA extraction, cDNA synthesis and qRT–PCR.

### RNAi

To achieve transient gene depletion, the RNAi feeding method was used^55,56^. Clones encoding the respective gene of interest were either taken from the *C. elegans* RNAi Collection (Ahringer) or the *C. elegans* ORF-RNAi Resource (Vidal)^57^, both distributed by Source BioScience. All clones were confirmed to contain the right gene by sequencing. All double knockdowns were performed with a single RNAi vector for high efficiency. In general, overnight cultures of transformed HT115 *E. coli* in LB medium with 100 µg ml^−1^ ampicillin were used as starting culture. A fresh culture was grown until an OD_600_ of approximately 0.8 and subsequently seeded on NGM agar plates containing 100 µg ml^−1^ ampicillin and 2 mM IPTG. Plates were incubated overnight at 37 °C and used for experiments. Age-synchronized worms were transferred to RNAi plates at L1 larval stage.

### Viral infection

Five adult worms were placed on an NGM plate. On the following day, worms were removed and hatched eggs were infected with 30 µl of Orsay viral lysate. Orsay lysate was obtained as previously described^25^. On the first day of adulthood, worms were either collected for western blotting of RNA extraction or worms were sorted for infection with the COPAS BioSorter (Union Biometrica) by using the viral reporter *Plys-3*::*gfp*^19^. To study the influence of ER stress on viral infection worms were pre-treated with either 5 µg ml^−1^ tunicamycin or DMSO (solvent control) for 8 h before viral infection.

### Cellular fractionation

For cellular fractionation, age-synchronized worms were washed three times with M9 buffer and stored in fractionation buffer (5 M NaCl, 1 M Na_2_CO_3_, 10% Triton X-100 or 1% SDS) at −80 °C. While proteins associated to ER-derived microsomes are solubilized by sodium chloride (NaCl) or sodium carbonate (Na_2_CO_3_), resident proteins inside the microsomes are released only by dissolving the ER membrane with detergents such as Triton X-100 or SDS. Samples were thawed on ice and sonicated for 20 s (60% amplitude) and homogenized using a Dounce homogenizer 50 times. Cell lysates were centrifuged two times for 5 min at 2,000 RCF at 4 °C. The supernatant was again centrifuged for 90 min at 20,000 RCF at 4 °C. The pellet was resuspended in 150 µl fractionation buffer, and 30 µl of the sample was supplemented with 3 µl fractionation buffer, respectively. Samples were incubated on ice for 1 h and subsequently centrifuged for 1 h at 20,000 RCF at 4 °C. Pellets and supernatants were separated and analysed via western blotting. Cellular fractionation using sequential detergent fractionation was performed as previously described^58^. Worm lysates were treated with the permeabilization buffer (110 mM KCl, 25 mM K-HEPES, pH 7.4, 1 mM MgCl_2_, 0.015% digitonin (Calbiochem), 0.1 mM EDTA, 40 units ml^−1^ RNAseOUT (Invitrogen) and 1 mM dithiothreitol) to release the cytosolic content. To release the ER fraction, worm lysates were further washed with ER fractionation buffer (2% dodecyl-maltoside, 200 mM KCl, 25 mM K-HEPES, pH 7.4, 10 mM MgCl_2_, 40 units ml^−1^ RNAseOUT and 2 mM dithiothreitol). Subsequently, samples were subjected to RNA extraction followed by qRT–PCR.

### Northern blotting

Denaturing RNA gel electrophoresis was performed using a 1% agarose gel (supplemented with 4% formaldehyde and 5% 20× TT (0.6 M tricine and 0.6 M triethanolamine). Total RNA was added to 16 µl of northern sample buffer (100 µl 20× TT, 4 µl 0.5 M EDTA, pH 8.0, 70 µl de-ionized formamide, 70 µl non-acidic formaldehyde (37%), 5 µl 0.07% ethidium bromide), heated for 15 min at 65 °C and immediately chilled on ice for 2 min. Before loading, 2 µl of RNA loading dye (1 mM EDTA, 50% glycerol and 0.25% bromophenol blue) was added to each sample. The gel was run at 150 V in 1× TT buffer for 3–4 h on ice. After RNA gel electrophoresis, 18S and 28S rRNA was imaged as gel loading control. Then, the separated RNA molecules were blotted on a neutral nylon membrane. After brief washing with H_2_O, the capillary transfer was set up for at least 4 h using 10× saline-sodium citrate (SSC) (1.5 M NaCl and 150 mM trisodium citrate, pH 7.0) as a transfer medium. Subsequently, the RNA was UV-crosslinked to the membrane at 254 nm (120 mJ cm^−2^, on both sides), followed by membrane pre-hybridization in Church buffer (0.5 M Na_2_HPO_4_, 1 mM EDTA and 7% SDS, pH 7.2) at 65 °C for 1 h. Further, the Church buffer was exchanged to 25 ml Church buffer supplemented with 25 µl ^32^P-labelled RNA probes directed against the corresponding sequences of the *cpl-*1 mRNA (5′-AGGAATGCTCATCCGAAGAGCTCGACCACGGAGTGCT

TCTCGTCGGATACGGAACCGACCCAGAGCACGGAGACTACTGGATTGT-3′) and the 5.8S rRNA (5′-CTAGCTTCAGCGATGGATCGGTTGCATCGAGTATCGATGAAGAACGCA

GCTTGCTGCGTTACTTACCACGAATTGCAGACGCTTAGAGTGGTGAAATTTCGAACGCATAGCACCAACTGGGCCTCCAGTTGGTACGTCTGGTTCAGGGTTGTT-3′). On the next day, the probe was removed from the membrane followed by washing twice with Northern wash buffer 1 (2× SSC and 0.1% SDS) and two wash steps with Northern wash buffer 2 (0.2× SSC and 0.1% SDS) at 65 °C for 15 min each. Subsequently, the membranes were air-dried for 40 min, covered with an imaging plate and exposed for 4 h at RT. The autoradiography signal was detected by using the Thyphoon FLA700 phosphoimager (GE Healthcare) and quantified with the ImageQuant TL, 1D and Array Image Analysis Software (GE Healthcare). For generation of the RNA probes, 150 ng linearized pGEM-4Z plasmid (Promega), containing the respective probe sequence, was in vitro transcribed using and SP6 RNA polymerase (New England Biolabs) according to the manufacturer’s instructions. The transcription reaction, supplemented with α-^32^P-GTP (Hartmann Analytics), was incubated for 40 min at 40 °C. Further, 1.5 µl RNAse-free DNAse I (2 units µl^−1^, New England Biolabs) was added, followed by incubation for 20 min at 37 °C. Subsequently, 28 µl ultrapure H_2_O was added and labelled transcripts were recovered using the mini-Quick Spin RNA Columns (Roche) following the manufacturer’s instructions. Finally, 25 µl of the probe was mixed with 25 ml Church buffer (1:1,000).

### RNA sequencing and analysis

Determination of mRNA expression was done by sequencing. Age-synchronized day 1 adult hermaphrodites were collected for isolation of total RNA from *C. elegans*. For cell culture experiments, *Ago2*^*−/−*^ and *Ago2*^*+/+*^ MEF cells were seeded into 150 cm^2^ flasks for collection of samples. After a 3 day growth (to around 70% confluency), either cells were left untreated or ER stress was induced by adding 3 µg ml^−1^ tunicamycin (Sigma-Aldrich) or 500 nM thapsigargin (Sigma-Aldrich) for 6 h. RNA isolation procedure was performed as described above. Total RNA (2 µg, 50–200 ng µl^−1^, OD_260/280_ 1.8–2.1 and OD_260/230_ >1.5) was sent to the Cologne Center of Genetics (University of Cologne). Quality control and sequencing was performed by the Cologne Center of Genetics. Raw reads were mapped to reference transcriptomes (Ensemble release 91) using HISAT2, and after guided transcriptome assembly with StringTie, quantification and differential expression was performed with Cufflinks. Calculation of *P* values in Cufflinks is performed with two-sided test for significance. In case a transcript has zero fragments in one condition, a one-sided test is performed. Cufflinks uses the Benjamini–Hochberg technique to correct for multiple testing. Hierarchical clustering of *z*-score-normalized FPKM (mean of each three replicates per strain or condition) was computed with R package ComplexHeatmap (version 2.4.2) with default settings^59^. Differential expression was visualized by scatterplots generated by ggplot2 (version 3.3.2). *z*-Score normalization and table organization were conducted with R Studio (version 4.0.3) using dplyr (version 1.0.2) and tidyr (1.1.0) packages. For GO analysis, GO PANTHER v.16.0 overrepresentation test was performed, with the respective ensemble gene IDs that were extracted from the UniProtKB UniRef90 database. We used the total list of transcripts in each experiment as the reference list for the GO PANTHER test. PANTHER calculates raw *P* values with the Fisher’s exact test and corrects for multiple comparison by the Benjamini–Hochberg procedure yielding the FDR value, which is displayed in Figs. 3b, 4f and 5d. *FDR <0.05; ** FDR <0.01; *** FDR <0.001; NS, FDR >0.05. The respective FASTA protein sequences were fetched from the UniProtKB UniRef90 database and were submitted to the BUSCA subcellular localization analysis tool^30^. All RNA sequencing, GO term and BUSCA analysis results are summarized in Supplementary Table 4.

### RNA–UV crosslink immunoprecipitation (RNA–CLIP)

For CLIP cell culture experiments *Ago2*^*−/−*^ and *Ago2*^*+/+*^ MEF cells were seeded into 150 cm^2^ flasks for collection of samples. After a 3 day growth (to around 70% confluency), cells were treated with tunicamycin (4 h, 3 µg ml^−1^, Sigma-Aldrich), UV-crosslinked (Stratalinker UV Crosslinker 1800), 150,000 µJ cm^−2^ on ice, washed in ice cold PBS, pelleted by centrifugation (5 min, 450 RCF) and stored at −80 °C. For immunoprecipitation (IP), the samples were thawed on ice and supplied with 1 ml of lysis buffer (50 mM Tris–HCl, 100 mM NaCl, 1% MP-40 (IGEPAL), 0.1% SDS and 0.5% sodium deoxycholate, pH 7.4) with freshly added protein inhibitor cocktail and RNase inhibitor according to the instructions of the manufacturer. The samples were resuspended in lysis buffer and lysed using a syringe with a 23 gauge and incubated 15 min on ice. The samples were sonicated in the Bioruptor (Diagenode) for 10 min with ten pulses of 30 s on and off. Two microlitres of TurboDNase (2 units µl^−1^, Thermo Fisher Scientific) was added, and the samples were shaken for 5 min at 37 °C and 1,200 rpm and subsequently centrifuged at 21,000 RCF for 15 min at 4 °C. After centrifugation, 2% of the total volume of the supernatant was put aside for RNA extraction and western blot analysis, respectively (input control, before IP). For the IP, 50 µl Dynabeads (Thermo Fisher Scientific) were coupled with 20 μl Ago2 antibody (Anti-Ago2 monoclonal (2D4), Fujifilm Wako Chemicals, catalogue number 014-22023) and rotated for 1 h at 4 °C. The coupled magnetic Dynabeads^®^ were then added to the samples and rotated overnight at 4 °C. The tubes were placed on the respective magnet tray to bind the magnet tubes at the side of the tube. Again, 20 μl of the supernatant was put aside for RNA extraction and western blot analysis, respectively (input control, after IP). The samples were washed three times with cold wash buffer (20 mM Tris–HCl, 10 mM MgCl_2_ and 0.2 Tween-20, pH 7.4) and resuspended in 100 μl wash buffer afterwards. Ten microlitres of each sample was put aside for western blot analysis, and 700 μl of TRIzol was added to the samples. RNA extraction was conducted with isopropanol–glycogen precipitation as described above, followed by qRT–PCR against the targets *Nov*, *Mertk*, *C1rl* and *Rpl19*. All antibodies that were used in this study are listed in Supplementary Table 2.

### Colony formation assay

We conducted a colony formation assay as described previously by Crowley et al.^60^. In brief, MEF cells were treated with tunicamycin for 4 h and collected by trypsinization. Treated cells were then seeded in six-well plates (800 cells per well) and incubated for 7 days. For staining of colonies, cells were first fixed with 100% methanol (20 min, RT) and rinsed with water. Then, colonies were stained with crystal violet solution (0.5% crystal violet (w/v) in 25% methanol) for 5 min at RT and again rinsed with water. Afterwards, plates were air-dried and imaged using a standard document scanner. Colonies were counted using the cell counter plugin of the ImageJ (1.48 v) software.

### Intestinal barrier function assay (Smurf assay)

Age-synchronized animals were grown on *E. coli* OP50 until day 5 of adulthood. On these days, about 30 worms were removed from NGM plates and washed once with M9 buffer. Animals were incubated rotating for 4 h at RT in liquid culture containing 100 µl blue food dye (50 µl 3× concentrated *E. coli* OP50 overnight culture in LB medium; 50 µl 5% w/v Brilliant Blue FCF (Sigma-Aldrich) in H_2_O) and 400 µl M9 buffer. After incubation, animals were washed twice with M9 buffer and collected by gravity settling. Subsequently, worms were transferred to agar pads on glass slides, paralysed with 25 mM levamisole (Sigma-Aldrich) and imaged with the Leica SCN400 slide scanner at 40x× magnification. Images were further processed using the Leica Aperio ImageScope software (v12.4.3.5008).

### Microscopy

*C. elegans* work was generally performed using the M80 stereomicroscope with a CLS 150× light source (Leica Camera). Fluorescent images were achieved using the M165 FC stereomicroscope with DFC 340 FX camera (Leica Camera) or the Axio Zoom V16 microscope with the Axiocam 506 mono camera (Carl Zeiss Microscopy GmbH). For imaging of ER structures in *C. elegans*, the confocal microscope TCS SP8 gSTED 3X (Leica Microsystems) was used. To immobilize worms for imaging, animals were treated with 25 mM levamisole (Sigma-Aldrich). Quantification of mCherry-HDEL foci was achieved with the ImageJ (1.48 v) software. If not stated otherwise, day 1 adult worms were imaged.

### Quantification, statistical analysis and reproducibility

Statistical details of experiments are described in the legends of the figures in which the relevant results are presented. Statistical analysis was performed using GraphPad Prism 5 and 7. In general, statistical significance was calculated with two-tailed paired Student’s *t*-test or one-way/two-way analysis of variance (ANOVA) with post-hoc test. The half-maximal inhibitory concentration (IC_50_) for tunicamycin treatment in MEF cells was determined with GraphPad Prism 7 using a three-parameter inhibitor versus response non-linear regression calculation (Fig. 5g). Unless stated otherwise, statistical analysis of BioSorter experiments was performed by two-sided pairwise *t*-test of mean values calculated on three independent biological experiments with each entailing at least 200 gated day 1 adult worms per experiment. *P* values were adjusted for multiple comparisons with Holmes method. All representative microscopy images and gel blots were performed in *n* = 3 independent experiments. No statistical method was used to pre-determine sample size. No data were excluded from the analyses. The experiments were not randomized, and the investigators were not blinded to allocation during experiments or outcome assessment.

### Reporting summary

Further information on research design is available in the Nature Portfolio Reporting Summary linked to this article.

## Online content

Any methods, additional references, Nature Portfolio reporting summaries, source data, extended data, supplementary information, acknowledgements, peer review information; details of author contributions and competing interests; and statements of data and code availability are available at https://doi.org/10.1038/s41556-022-01025-4.

## Supplementary information

Reporting Summary

Supplementary Tables *C. elegans*, bacterial strains, cell lines, antibodies and oligos used in this study (Supplementary Tables 1–3). Data from RNA-sequencing and bioinformatic analysis (Supplementary Table 4).

## Source data

Source Data Fig. 1 Uncropped blots.

Source Data Fig. 2 Statistical source data.

Source Data Fig. 2 Uncropped blots.

Source Data Fig. 3 Statistical source data.

Source Data Fig. 4 Statistical source data.

Source Data Fig. 5 Statistical source data.

Source Data Fig. 5 Uncropped blots.

Source Data Fig. 6 Statistical source data.

Source Data Fig. 6 Uncropped blots.

Source Data Extended Data Fig. 1 Uncropped blots.

Source Data Extended Data Fig. 2 Uncropped blots.

Source Data Extended Data Fig. 2 Statistical source data.

Source Data Extended Data Fig. 3 Statistical source data.

Source Data Extended Data Fig. 4 Statistical source data.

Source Data Extended Data Fig. 5 Uncropped blots.

Source Data Extended Data Fig. 5 Statistical source data.

Source Data Extended Data Fig. 6 Uncropped blots.

Source Data Extended Data Fig. 6 Statistical source data.

## Data Availability

Plasmids and *C. elegans* lines generated in this study will be distributed to other researchers upon request. RNA-sequencing data that support the findings of this study have been deposited in the Gene Expression Omnibus (GEO) under accession codes GSE202291 and GSE161121. Source data are provided with this paper. All other data supporting the findings of this study are available from the corresponding author on reasonable request. The UniProtKB UniRef90 database was used for GO PANTHER and BUSCA bioinformatic analysis.
