# Supplementary material for: ER-associated RNA silencing promotes ER quality control
Source: Nat Cell Biol. 2022 Dec 5;24(12):1714–25. doi: 10.1038/s41556-022-01025-4 (PMC9729107; doi:10.1038/s41556-022-01025-4)
Supplement: Supplementary file 1 — Reporting Summary [file 41556_2022_1025_MOESM1_ESM.pdf]

nature portfolio

Corresponding author(s): Prof. Dr. Thorsten Hoppe

Last updated by author(s): 28.09.2022

Reporting Summary

Nature Portfolio wishes to improve the reproducibility of the work that we publish. This form provides structure for consistency and transparency in reporting. For further information on Nature Portfolio policies, see our Editorial Policies and the Editorial Policy Checklist.

Statistics

For all statistical analyses, confirm that the following items are present in the figure legend, table legend, main text, or Methods section.

n/a	Confirmed	
☐	☒	The exact sample size (*n*) for each experimental group/condition, given as a discrete number and unit of measurement
☐	☒	A statement on whether measurements were taken from distinct samples or whether the same sample was measured repeatedly
☐	☒	The statistical test(s) used AND whether they are one- or two-sided<br>*Only common tests should be described solely by name; describe more complex techniques in the Methods section.*
☒	☐	A description of all covariates tested
☐	☒	A description of any assumptions or corrections, such as tests of normality and adjustment for multiple comparisons
☐	☒	A full description of the statistical parameters including central tendency (e.g. means) or other basic estimates (e.g. regression coefficient) AND variation (e.g. standard deviation) or associated estimates of uncertainty (e.g. confidence intervals)
☐	☒	For null hypothesis testing, the test statistic (e.g. *F*, *t*, *r*) with confidence intervals, effect sizes, degrees of freedom and *P* value noted<br>*Give P values as exact values whenever suitable.*
☒	☐	For Bayesian analysis, information on the choice of priors and Markov chain Monte Carlo settings
☒	☐	For hierarchical and complex designs, identification of the appropriate level for tests and full reporting of outcomes
☒	☐	Estimates of effect sizes (e.g. Cohen's *d*, Pearson's *r*), indicating how they were calculated

Our web collection on statistics for biologists contains articles on many of the points above.

Software and code

Policy information about availability of computer code

Data collection	Image Studio Software v4.0 or v5.0, LI-COR Biosciences<br>Bio-Rad CFX Manager Software 3.1<br>FlowPilot-Pro™ (v1)

Data analysis

Image Studio Software v4.0 or v5.0, LI-COR Biosciences
Bio-Rad CFX Manager Software 3.1
FlowPilot-Pro™ (v1)
ImageQuant TL 10.2 (GE Healthcare)
GO PANTHER v.16.0
ImageJ (1.48v)
Leica Aperio ImageScope software v12.4.3.5008

R Studio (version 4.0.3)
R pipelines HISAT2, StringTie and Cufflinks
R package ComplexHeatmap (version 2.4.2)
R package ggplot2 (version 3.3.2).
R package dplyr (version 1.0.2)
R package tidyr (1.1.0)
R package ggplot2 v.3.6.2
R package ggridges v.3.6.2
R package hexbin v.1.28.2

For manuscripts utilizing custom algorithms or software that are central to the research but not yet described in published literature, software must be made available to editors and reviewers. We strongly encourage code deposition in a community repository (e.g. GitHub). See the Nature Portfolio guidelines for submitting code & software for further information.

Data

Policy information about availability of data

All manuscripts must include a data availability statement. This statement should provide the following information, where applicable:

- Accession codes, unique identifiers, or web links for publicly available datasets
- A description of any restrictions on data availability
- For clinical datasets or third party data, please ensure that the statement adheres to our policy

The authors declare that the main data supporting the findings of this study are available within the article, its Supplementary Information files or in public repositories, which can be accessed with the provided links. Extra data are available from the corresponding author upon request.

Plasmids and C. elegans lines generated in this study will be distributed to other researchers upon request. RNA–sequencing data that support the findings of this study have been deposited in the Gene Expression Omnibus (GEO) under accession codes GSE202291 and GSE161121. Source data have been provided in Source Data. All other data supporting the findings of this study are available from the corresponding author on reasonable request. The UniProtKB UniRef90 database was used for GO PANTHER and BUSCA bioinformatic analysis.

Human research participants

Policy information about studies involving human research participants and Sex and Gender in Research.

Reporting on sex and gender

Use the terms sex (biological attribute) and gender (shaped by social and cultural circumstances) carefully in order to avoid confusing both terms. Indicate if findings apply to only one sex or gender; describe whether sex and gender were considered in study design whether sex and/or gender was determined based on self-reporting or assigned and methods used. Provide in the source data disaggregated sex and gender data where this information has been collected, and consent has been obtained for sharing of individual-level data; provide overall numbers in this Reporting Summary. Please state if this information has not been collected. Report sex- and gender-based analyses where performed, justify reasons for lack of sex- and gender-based analysis.

Population characteristics

Describe the covariate-relevant population characteristics of the human research participants (e.g. age, genotypic information, past and current diagnosis and treatment categories). If you filled out the behavioural & social sciences study design questions and have nothing to add here, write "See above."

Recruitment

Describe how participants were recruited. Outline any potential self-selection bias or other biases that may be present and how these are likely to impact results.

Ethics oversight

Identify the organization(s) that approved the study protocol.

Note that full information on the approval of the study protocol must also be provided in the manuscript.

Field-specific reporting

Please select the one below that is the best fit for your research. If you are not sure, read the appropriate sections before making your selection.

☒ Life sciences ☐ Behavioural & social sciences ☐ Ecological, evolutionary & environmental sciences

For a reference copy of the document with all sections, see nature.com/documents/nr-reporting-summary-flat.pdf

Life sciences study design

All studies must disclose on these points even when the disclosure is negative.

Sample size	Sample size determination was done according to standard C. elegans approaches. Exact sample sizes are stated in the according figure legends and Supplementary Information. No statistical method was used to predetermine sample size. Overall at least three biological replicates were performed for each experiment, which is an established norm in the scientific community.
Data exclusions	No data were excluded from the analyses.
Replication	Except for Figure S5f, at least three biological replicates were performed for each experiment.
Randomization	The experiments were not randomized by purpose. Randomization was not applicable in our studies since C.elegans population are inherently isogenic and their distribution to different treatment regimes is per se random.
Blinding	The Investigators were not blinded to allocation during experiments and outcome assessment. All experiments in the study were quantified by automated and objective readout methods for outcome assesment (RT-qPCR, measuring fluorescence, fluorescent foci, etc.).

Reporting for specific materials, systems and methods

We require information from authors about some types of materials, experimental systems and methods used in many studies. Here, indicate whether each material, system or method listed is relevant to your study. If you are not sure if a list item applies to your research, read the appropriate section before selecting a response.

Materials & experimental systems

n/a	Involved in the study	
☐	☒	Antibodies
☐	☒	Eukaryotic cell lines
☒	☐	Palaeontology and archaeology
☐	☒	Animals and other organisms
☒	☐	Clinical data
☒	☐	Dual use research of concern

Methods

n/a	Involved in the study	
☒	☐	ChIP-seq
☐	☒	Flow cytometry
☒	☐	MRI-based neuroimaging

Antibodies

Antibodies used	Mouse monoclonal anti alpha tubulin (clone B-5-1-2) Sigma Cat# T6074, RRID:AB_477582, dilution 1:5000;<br>Mouse monoclonal anti Living Colors, (clone JL-8), (anti-GFP) Clontech Laboratories, Inc. Cat# 632380, RRID:AB_10013427, dilution1:5000;<br>Mouse monoclonal anti Living Colors, DsRed, (anti-mCherry; no clone name provided by Clonetech Laboratories) Clontech Laboratories, Inc. Cat# 632393, RRID:AB_2801258, dilution 1:5000;<br>Anti-CDC-48.1, Hoppe-lab/Biogenes Berlin, custom antibody, dilution 1:50000;<br>Anti-SEL-1, Sommer-lab, Berlin, custom antibody, dilution 1:8000;<br>Anti-Ago2 monoclonal (C34C6)(western bloting), Cell Signaling Cat# 2897, RRID:AB_2096291, dilution 1:10000;<br>Anti-Ago2 monoclonal (2D4)(UV-crosslinking immunoprecipitation), Fujifilm Wako Chemicals, Cat# 014-22023, RRID:AB_1106837, dilution: 20 µl per CLIP reaction;<br>Anti-alpha (orsay virus) Wang-lab, Washington, custom antibody, dilution 1:2000;<br>IRDye® 800CW Donkey anti-Mouse IgG (H + L); LI-COR Biosciences, Cat# 926-32212, RRID:AB_2716622, dilution 1:10000;<br>IRDye® 800CW Donkey anti-Rabbit IgG (H + L), LI-COR Biosciences, Cat# 926-32213, RRID:AB_621848, dilution 1:10000;<br>IRDye® 680 Donkey anti-Mouse IgG (H + L), LI-COR Biosciences, Cat# 962-32222, RRID:AB_621844, dilution 1:10000;<br>IRDye® 680 Donkey anti-Rabbit IgG (H + L), LI-COR Biosciences, Cat# 962-32223, RRID:AB_621845, dilution 1:10000
Validation	Validations of primary and secondary antibodies were done by the stated manufacturer's or by the laboratory they where produced by.<br>Anti-CDC-48.1, Hoppe-lab/Biogenes Berlin, custom antibody: Franz et al., Mol. Cell., 2014<br>Anti-SEL-1, Jarosch-lab, Berlin, custom antibody: Denzel et al., Cell, 2014<br>Anti-alpha (orsay virus) Wang-lab, Washington, custom antibody: Jiang et al., Virology, 2014<br><br>Mouse monoclonal anti alpha tubulin (clone B-5-1-2):<br>Independent Antibody Verification – Demonstrating antibody specificity through the use of multiple antibodies against target in IHC or ICC. (https://www.sigmaaldrich.com/DE/de/technical-documents/technical-article/protein-biology/immunohistochemistry/antibody-enhanced-validation)<br><br>Mouse monoclonal anti Living Colors, (JL-8), (anti-GFP):<br>The quality and performance of this lot of Living Colors A.v. Monoclonal Antibody (JL-8) was tested by Western blot

analysis using lysate made from a HEK 293 cell line stably expressing AcGFP1. After cells were collected and lysed using SDS sample buffer, the lysate (10 µl; equivalent to 35,000 cells) was electrophoresed on a 12% SDSpolyacrylamide gel and transferred to a nitrocellulose membrane. The blot was probed with the Living Colors A.v. Monoclonal Antibody, JL-8 (diluted 1:1,000), followed by a secondary goat anti-mouse antibody conjugated to horseradish peroxidase (HRP). The HRP signal was detected by chemiluminescence. A band of approximately 30 kDa corresponding to AcGFP1 was observed in the lane loaded with the AcGFP1 cell lysate. A band of this size was not detected in the lysate of untransfected HEK 293 cells.

Living Colors® DsRed Monoclonal Antibody:
The quality and performance of this lot of antibody was tested by Western blot analysis using lysate made from a HEK 293 cell line stably expressing DsRed Express. After cells were collected and lysed using SDS sample buffer, the lysate (10 µl; equivalent to 35,000 cells) was electrophoresed on a 12% SDS-polyacrylamide gel and transferred to a nitrocellulose membrane.The blot was probed with the Living Colors DsRed MonoclonalAntibody (diluted 1:500), followed by a secondary goat anti-mouse antibody conjugated to horseradish peroxidase (HRP). The HRP signal was detected by chemiluminescence. A band of approximately 29 kDa corresponding to DsRed Express was observed in the lane loaded with the DsRed Express cell lysate. A band of this size was not detected in the lysate of untransfected HEK 293 cells.

Anti-Ago2 monoclonal (western blotting) (C34C6):
No rating or validation information has been found for Rabbit Anti-Argonaute 2 Monoclonal Antibody, Unconjugated, Clone C34C6.

Anti-Ago2 monoclonal (UV-CLIP) (2D4):
No rating or validation information has been found for Anti-Argonaute 2 Monoclonal Antibody (2D4)

Eukaryotic cell lines

Policy information about cell lines and Sex and Gender in Research

Cell line source(s)	Ago2+/+ and Ago-/- Mouse embryonic fibroblasts were recieved from Greg Hannon, Cambridge, UK.
Authentication	None of the cell lines used in this study were authenticated. However, Ago2-/- MEF cells were tested for absence of Ago2 via western blot.
Mycoplasma contamination	All cell lines used in this study were regularly tested negative for Mycoplasma contamination.
Commonly misidentified lines (See ICLAC register)	According to the ICLAC register, no commonly misidentified cell lines were used in this study.

Animals and other research organisms

Policy information about studies involving animals; ARRIVE guidelines recommended for reporting animal research, and Sex and Gender in Research

Laboratory animals	The study involved Caenorhabditis elegans strains of various genotypes listed in the provided Supplementary Information. C. elegans populations entail predominantly hermaphrodites. The age of the tested organisms is described in the respective method section.
Wild animals	The study did not involve wild animals.
Reporting on sex	All experiments in this study were performed on hermaphrodite C. elegans populations.
Field-collected samples	The study did not involve samples collected from the field.
Ethics oversight	This study only includes work with the nematode C. elegans or with Mouse embryonic fibroblasts. For both no ethical approval or guidance is required.

Note that full information on the approval of the study protocol must also be provided in the manuscript.

Flow Cytometry

Plots

Confirm that:

☒ The axis labels state the marker and fluorochrome used (e.g. CD4-FITC).

☒ The axis scales are clearly visible. Include numbers along axes only for bottom left plot of group (a 'group' is an analysis of identical markers).

☒ All plots are contour plots with outliers or pseudocolor plots.

☒ A numerical value for number of cells or percentage (with statistics) is provided.

Methodology

Sample preparation	1000 age-synchronized worms were grown on L-plates until day 1 adulthood. Worms were washed off in 10 ml of M9 buffer.
Instrument	BioSorter® (Union Biometrica)
Software	FlowPilot-Pro™ (v1)
Cell population abundance	1000 worms per sample
Gating strategy	Unless stated otherwise, gating was performed with TOF >1500, Red >300, Extinction >800. Experiments with smaller sel-11(nDf59) mutant worms were gated with (TOF >1200, Red >300, Extinction >600). Only gated worms were quantified for the fluorescence of interest.

☒ Tick this box to confirm that a figure exemplifying the gating strategy is provided in the Supplementary Information.
