# Supplementary figures and images for: ER-associated RNA silencing promotes ER quality control
Source: Nat Cell Biol. 2022 Dec 5;24(12):1714–25. doi: 10.1038/s41556-022-01025-4 (PMC9729107; doi:10.1038/s41556-022-01025-4)

uncropped blots related for Fig. 1:

**C**

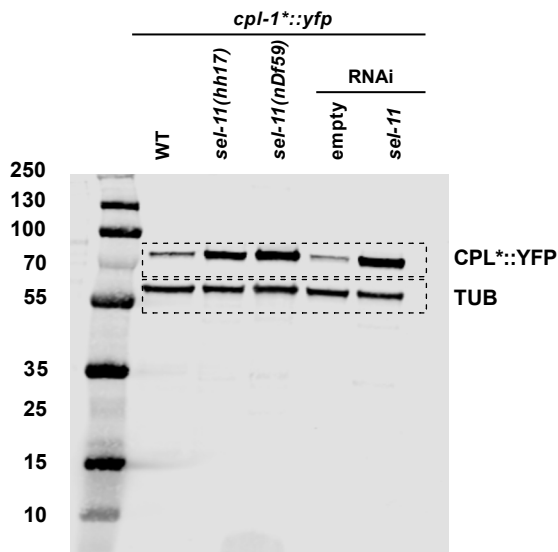

**E**

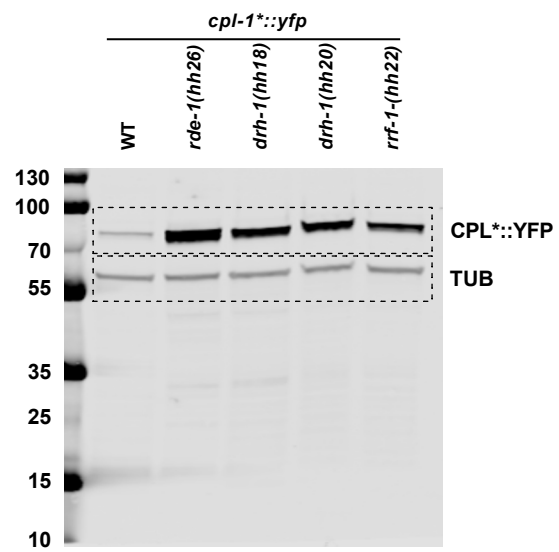

**F**

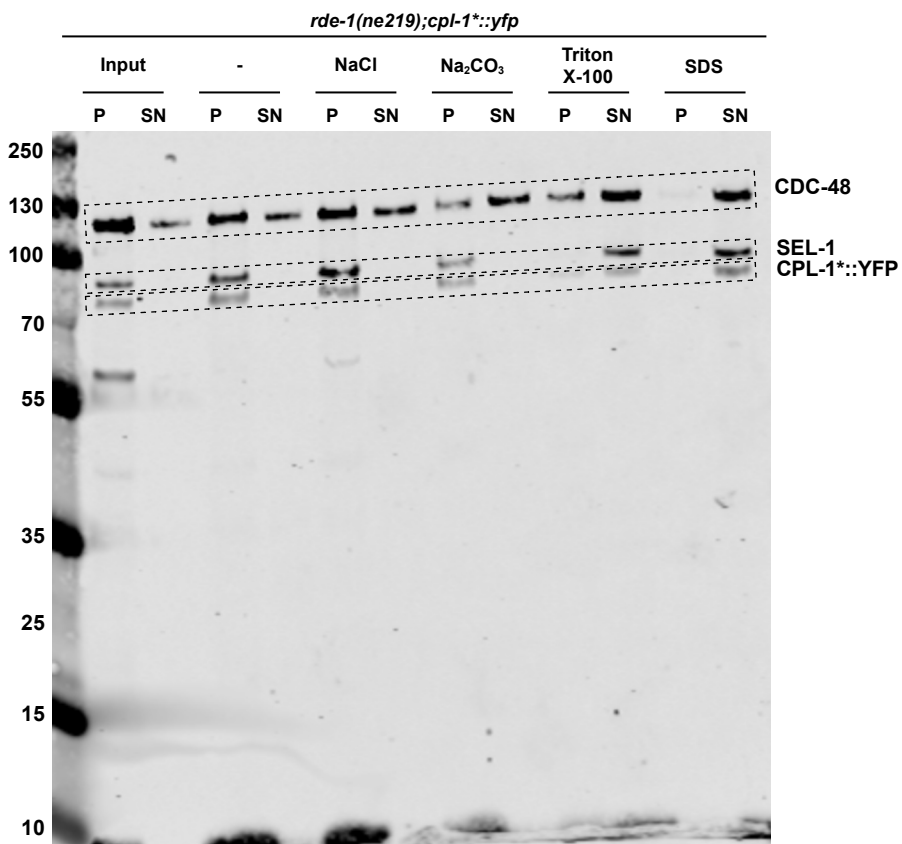

**G**

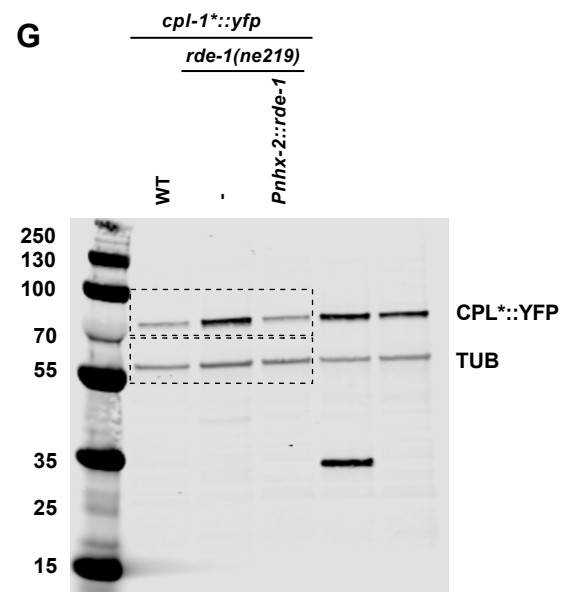

Supplement: Source Data Fig. 1 — Uncropped blots. [file 41556_2022_1025_MOESM3_ESM.pdf]

uncropped blots related to Figure 2:

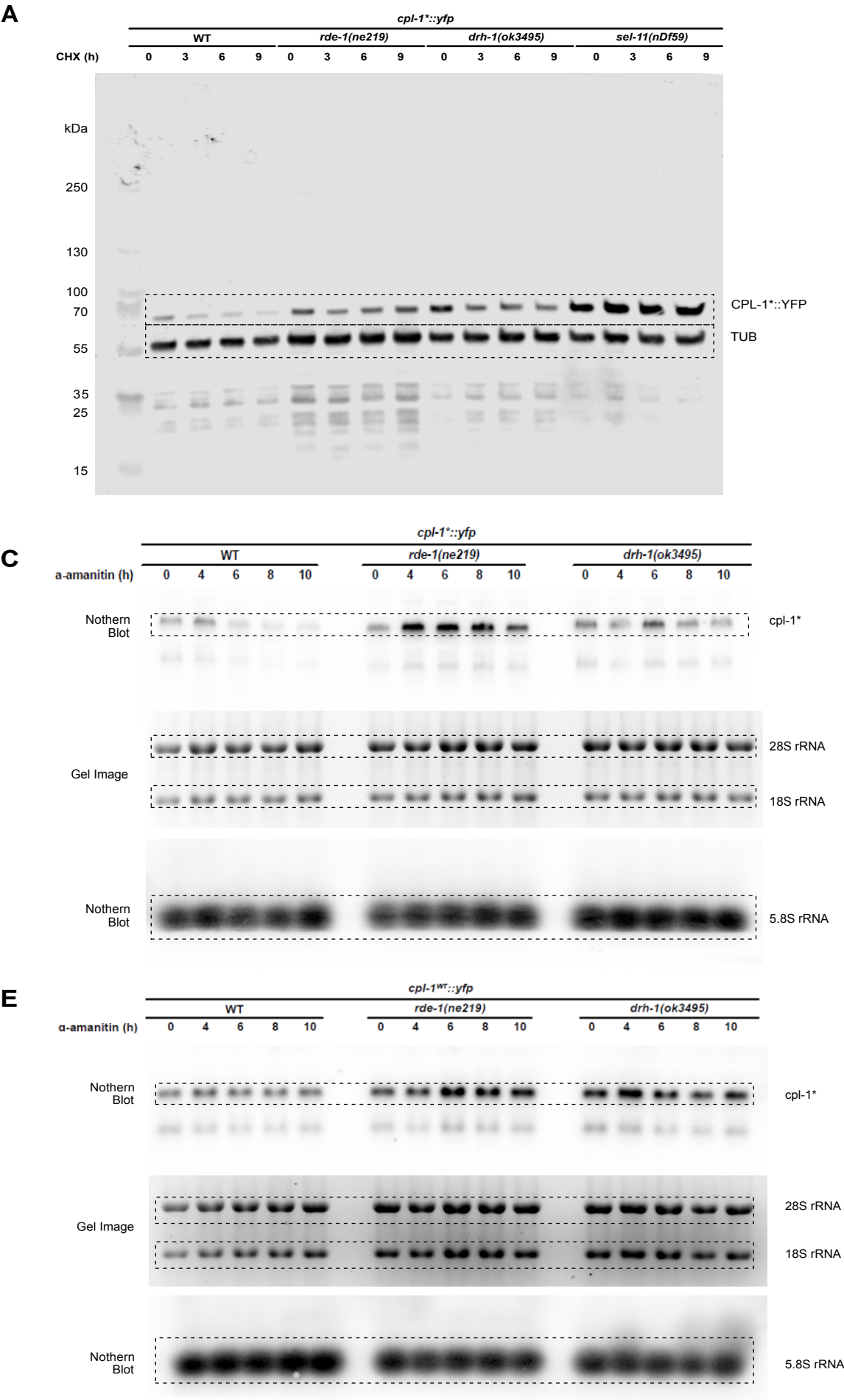

Supplement: Source Data Fig. 2 — Uncropped blots. [file 41556_2022_1025_MOESM5_ESM.pdf]

uncropped blots related to Fig. 5:

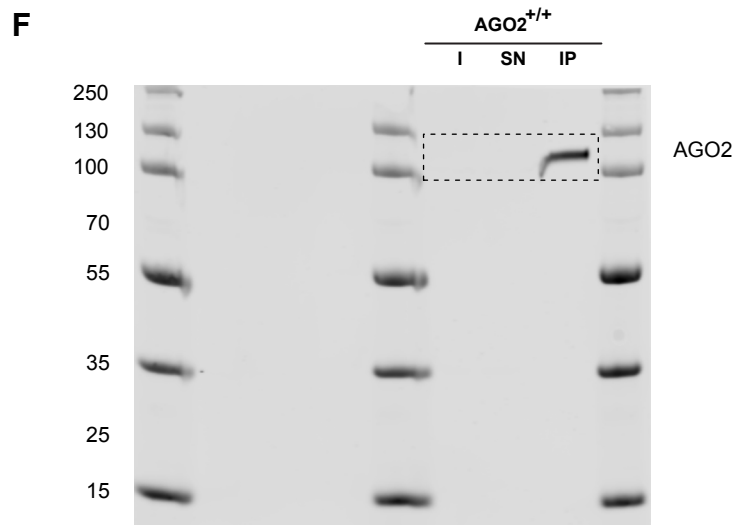

Supplement: Source Data Fig. 5 — Uncropped blots. [file 41556_2022_1025_MOESM9_ESM.pdf]

uncropped blots related to Fig. 6:

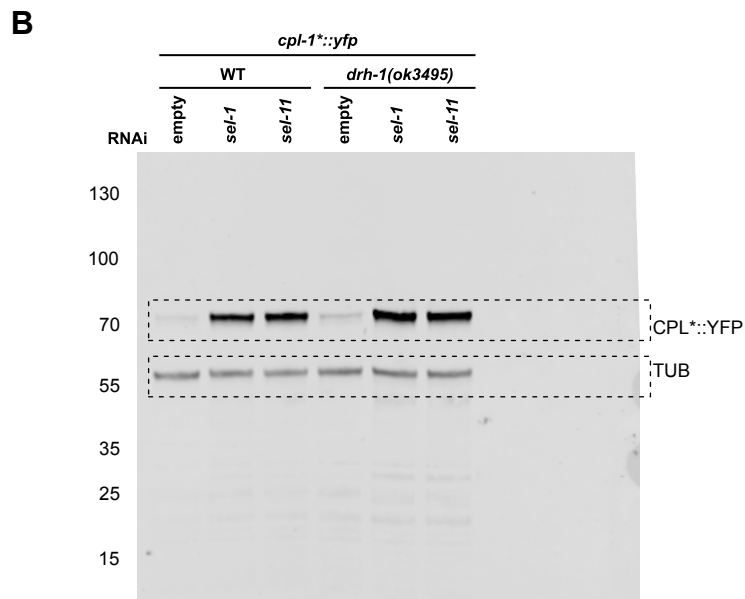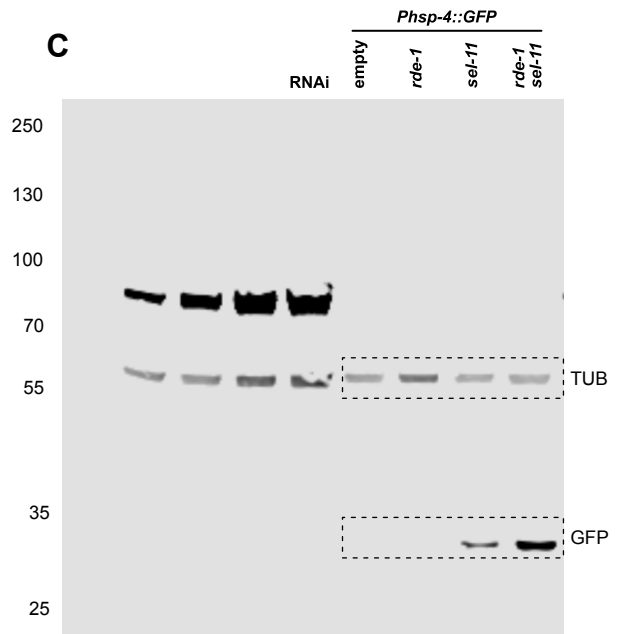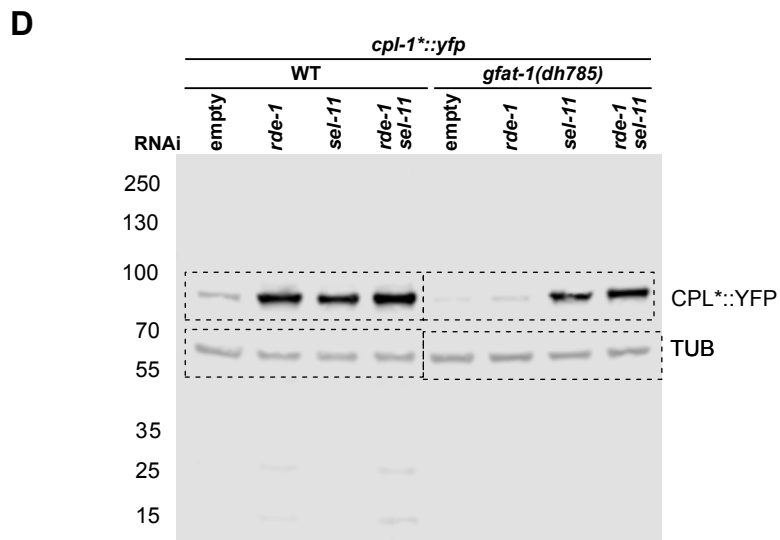

Supplement: Source Data Fig. 6 — Uncropped blots. [file 41556_2022_1025_MOESM11_ESM.pdf]

uncropped blots related to Fig. S1:

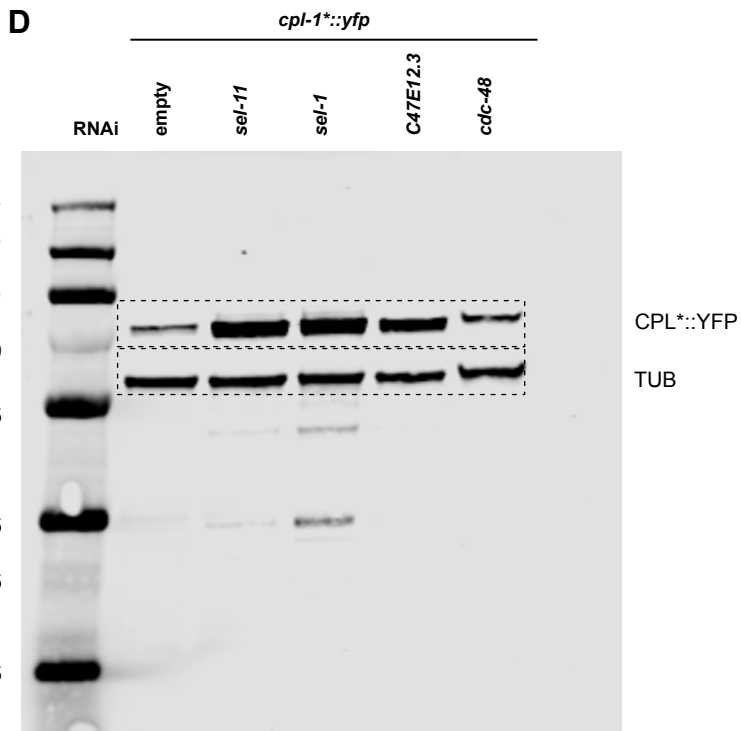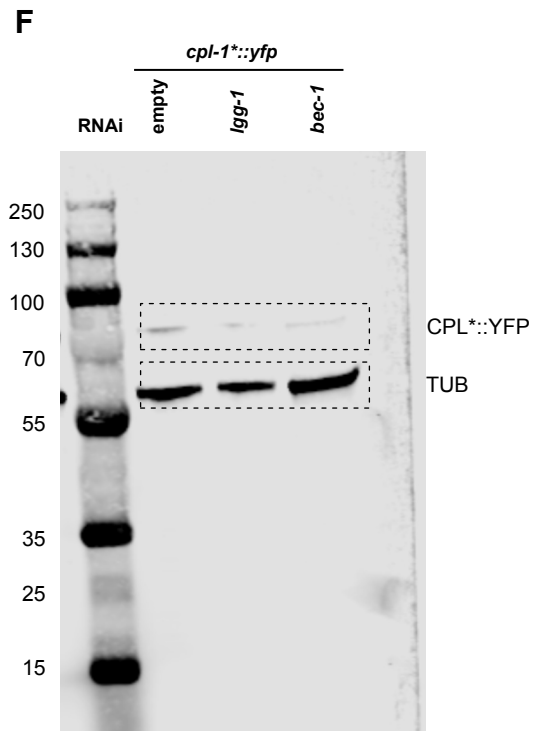

Supplement: Source Data Extended Data Fig. 1 — Uncropped blots. [file 41556_2022_1025_MOESM12_ESM.pdf]

uncropped blots related to Fig. S2:

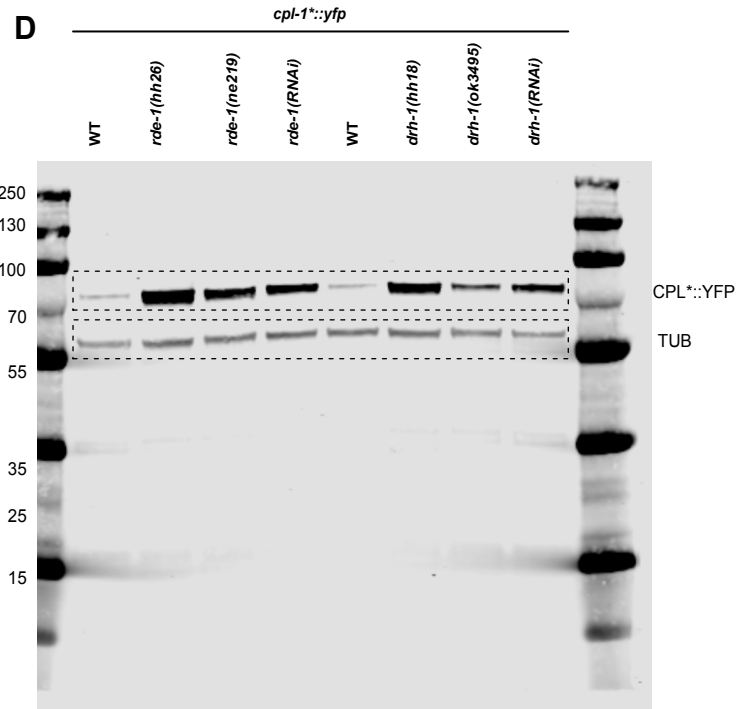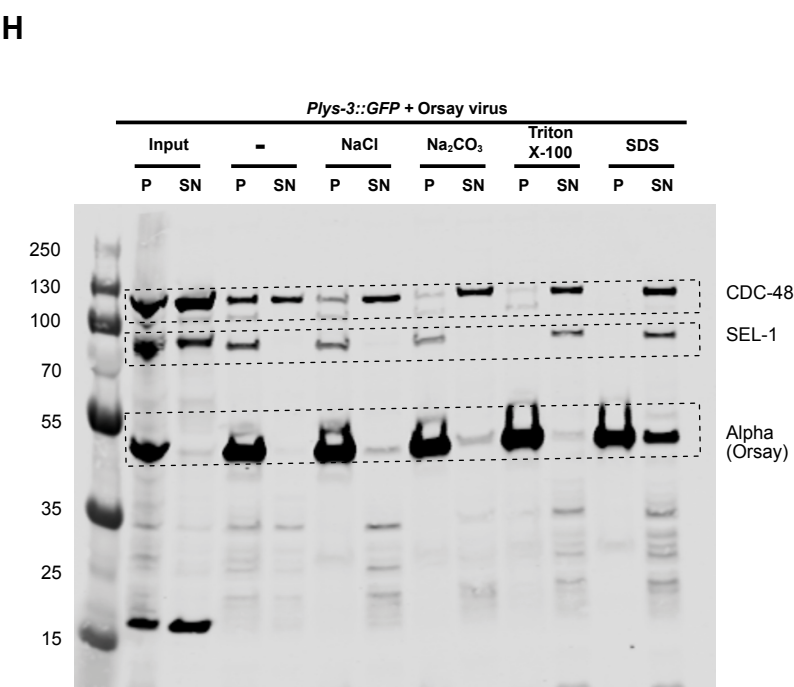

Supplement: Source Data Extended Data Fig. 2 — Uncropped blots. [file 41556_2022_1025_MOESM13_ESM.pdf]

uncropped blots related to Fig. S5:

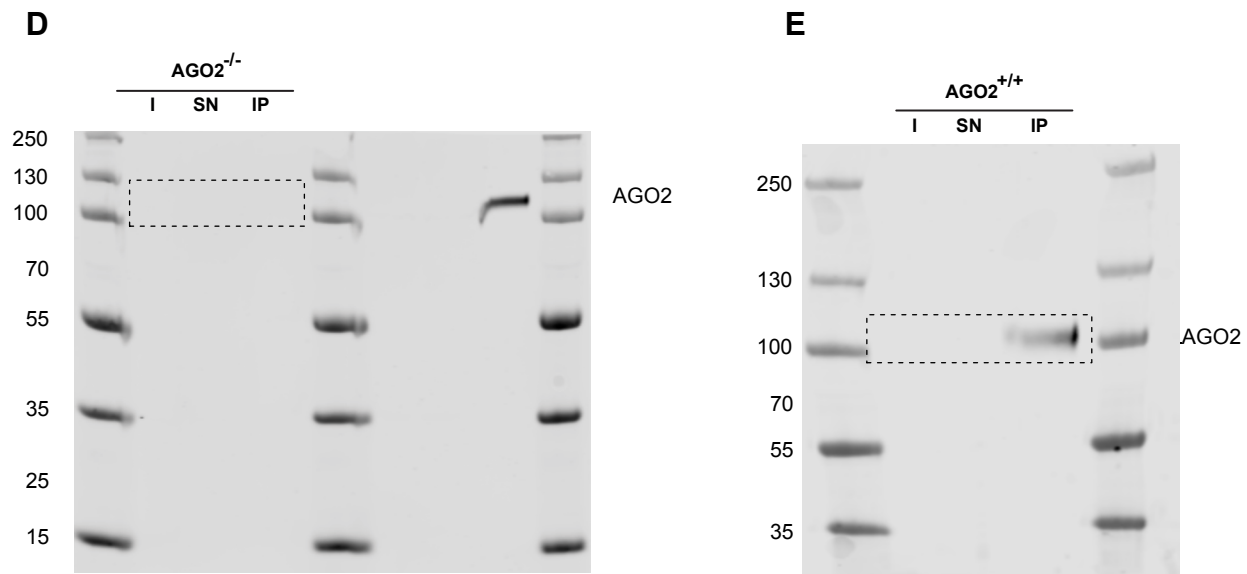

Supplement: Source Data Extended Data Fig. 5 — Uncropped blots. [file 41556_2022_1025_MOESM17_ESM.pdf]

uncropped blots related to Fig. S6:

**A**

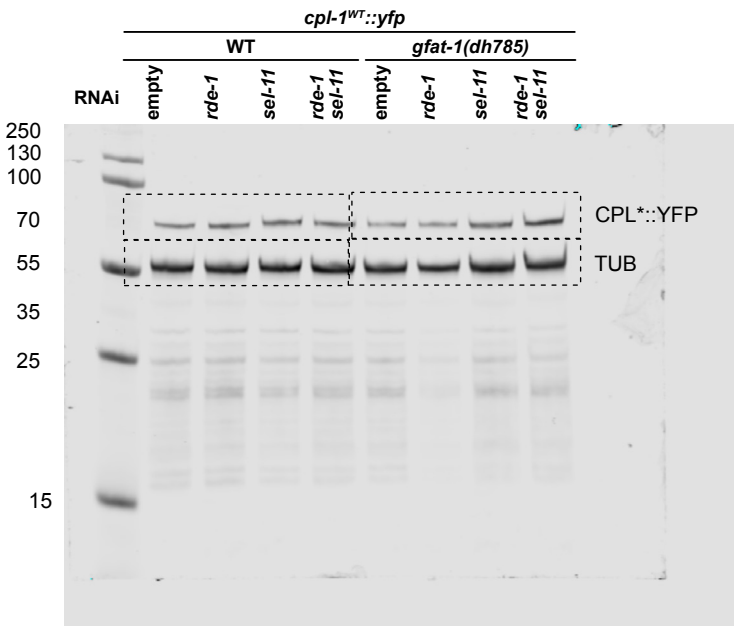

**D**

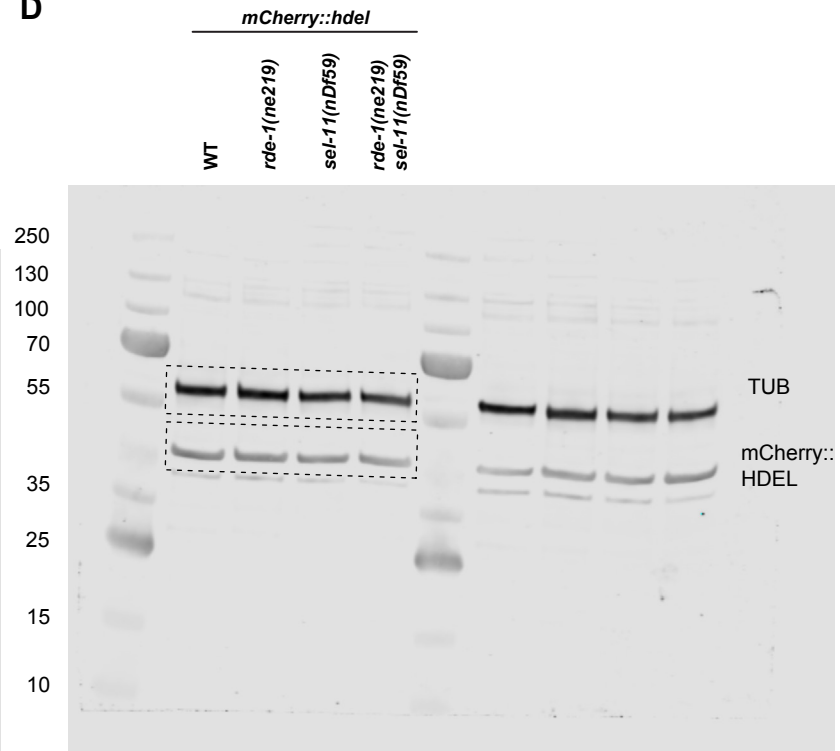

Supplement: Source Data Extended Data Fig. 6 — Uncropped blots. [file 41556_2022_1025_MOESM19_ESM.pdf]
